# Patterned Vascularization of Embryonic Mouse Forebrain, and Neuromeric Topology of Major Human Subarachnoidal Arterial Branches: A Prosomeric Mapping

**DOI:** 10.3389/fnana.2019.00059

**Published:** 2019-06-19

**Authors:** Luis Puelles, Rafael Martínez-Marin, Pedro Melgarejo-Otalora, Abdelmalik Ayad, Antonios Valavanis, José Luis Ferran

**Affiliations:** ^1^Department of Human Anatomy, School of Medicine, University of Murcia and IMIB-Arrixaca Institute, Murcia, Spain; ^2^Department of Neuroradiology, University Hospital of Zurich, Zurich, Switzerland

**Keywords:** brain arteries, penetrating vessels, arterial topology, arterial branching, terminal fields, molecular profile

## Abstract

The prosomeric brain model contemplates progressive regionalization of the central nervous system (CNS) from a molecular and morphological ontogenetic perspective. It defines the forebrain axis relative to the notochord, and contemplates intersecting longitudinal (zonal, columnar) and transversal (neuromeric) patterning mechanisms. A checkboard pattern of histogenetic units of the neural wall results, where each unit is differentially fated by an unique profile of active genes. These natural neural units later expand their radial dimension during neurogenesis, histogenesis, and correlative differential morphogenesis. This fundamental topologic framework is shared by all vertebrates, as a Bauplan, each lineage varying in some subtle aspects. So far the prosomeric model has been applied only to neural structures, but we attempt here a prosomeric analysis of the hypothesis that major vessels invade the brain wall in patterns that are congruent with its intrinsic natural developmental units, as postulated in the prosomeric model. Anatomic and embryologic studies of brain blood vessels have classically recorded a conserved pattern of branches (thus the conventional terminology), and clinical experience has discovered a standard topography of many brain arterial terminal fields. Such results were described under assumptions of the columnar model of the forebrain, prevalent during the last century, but this is found insufficient in depth and explanatory power in the modern molecular scenario. We have thus explored the possibility that brain vascularization in rodents and humans may relate systematically to genoarchitectonic forebrain subdivisions contemplated in the prosomeric model. Specifically, we examined first whether early vascular invasion of some molecularly characterized prosomeric domains shows heterochrony. We indeed found a heterochronic pattern of vascular invasion that distinguishes between adjacent brain areas with differential molecular profiles. We next mapped topologically on the prosomeric model the major arterial branches serving the human brain. The results of this approach bear on the possibility of a developmentally-based modern arterial terminology.

## Introduction

Once development of the closed neural tube progresses beyond patterning, regionalization and initial surface growth, the processes of neurogenesis and differentiation commence in an heterochronic pattern, showing gradual construction of a heterogeneous mantle layer. According to its state of differential histogenetic specification, each progenitor domain is programmed to produce characteristic neuronal populations, whose identity is now largely known by molecular maps and fate mapping experiments (Puelles et al., 1987, 2000; Cobos et al., 2001; García-López et al., 2004, 2009; Pombero and Martínez, 2009; Puelles and Ferran, 2012). Generation of immature mantle strata (pronuclei) and definitive nuclei or layers of each cerebral region is closely correlated with the acquisition of a network of penetrating and internally ramifying blood vessels which supply the metabolites demanded by the growing tissue (James and Mukouyama, 2011).

**Figure 1:**
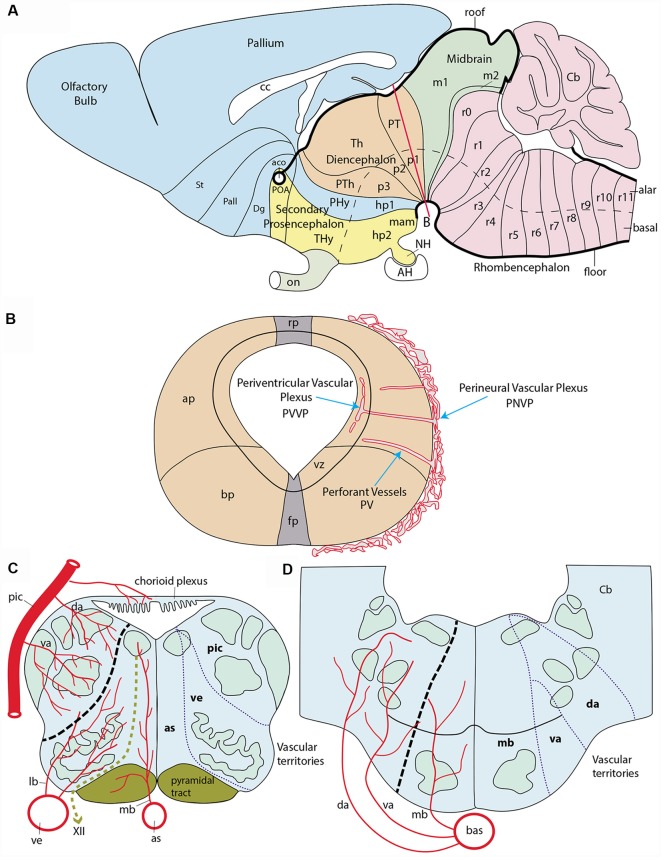
Schematic introduction to topologic mapping of brain arteries, based on the rodent brain. **(A)** Lateral view of updated prosomeric model showing color-coded forebrain and hindbrain regions, subdivided into neuromeric units (pink = hindbrain, r0-r11; green = midbrain, m1, m2; cream = diencephalon, p1-p3; blue = first hypothalamo-telencephalic prosomere, hp1, contains peduncular hypothalamus, PHy, and evaginated telencephalon, with pallium and subpallial subdivisions St, Pall, Dg; yellow = second hypothalamo-telencephalic prosomere, hp2, contains terminal hypothalamus, THy, and unevaginated subpallial preoptic area, POA). The roof and floor plates are marked with thick black lines. Note convergence of transverse interneuromeric boundaries at the cephalic flexure, due to axial bending of the forebrain. The red line finishing near the big letter **(B)** represents a transversal plane of section through the p1 neuromere. **(B)** Schematic cross-section at the level marked by red line in **(A)**. It illustrates the main vascularization steps. The fundamental longitudinal zones, floor, basal, alar and roof plates (fp, bp, ap, rp) are displayed jointly with the alar-basal boundary. Early brain-invading blood vessels form a perineural vascular plexus (PNVP), perforant vessels (PV) and a deep periventricular vascular plexus (PVVP; note the PVVP actually lies within the proliferative ventricular zone, rather than periventricularly). **(C)** Schema of main basal and alar arterial vessels in the human hindbrain medulla. The black dash line at left marks the alar-basal boundary, while the green dash line identifies the hypoglossal nerve root. Direct penetrating mediobasal and laterobasal branches (mb, lb) originate respectively from the longitudinal anterior spinal (as) or vertebral (ve) arteries, while arteries serving the alar plate, representing so-called short or long circumferential vessels, distinguish ventral and dorsal levels of this domain. We identify them as ventroalar (va) and dorsoalar (da) arteries. At this particular level the va and da branches originate from the postero-inferior cerebellar artery (pic), but otherwise, they each originate directly from the basilar artery. The respective as, ve (basal) and pic (alar) dependent fields are delimited at right. **(D)** Schema of main basal and alar arterial vessels in the human hindbrain pons. The black dash line at left marks the alar-basal boundary. Mediobasal (mb) as well as ventrolar and dorsoalar (va, da) arteries arise as lateral branches of the basilar artery (bas) and penetrate radially their respective basal and alar terminal fields, delineated at the right side.

The development of the central nervous system (CNS) wall is a stereotyped regionalization process, orchestrated by diverse signaling molecules spreading gradientally from primary and secondary organizers. Intersecting anteroposterior (AP) and dorsoventral (DV) patterning effects taking place during early brain regionalization specify primary cerebral compartments, as well as secondary subdivisions. These display a checkboard pattern of orthogonal boundaries (AP patterning produces transverse segments or neuromeres, separated by interneuromeric boundaries, whereas DV patterning produces longitudinal zones). This establishes already at early neuroepithelial stages a checkered fundamental plan of construction of the neural tube wall (a brain Bauplan), which is apparently shared among all vertebrates (Nieuwenhuys and Puelles, 2016). The basic details of this neuromeric and longitudinal Bauplan have been recently encapsulated by the *prosomeric model* (Figure 1A; Puelles and Rubenstein, 1993, 2003, 2015; Puelles et al., 2013; Puelles, 2013). Note the historically earlier *columnar model* (Herrick, 1910; Kuhlenbeck, 1973; Swanson, 2012) attended essentially to longitudinal subdivisions—e.g., “brain columns,”—but disregarded transversal units other than the major brain vesicles. This feature, jointly with an arbitrarily-defined forebrain axis, eventually caused its present insufficiency as a brain model.

According to the prosomeric model, the transverse neuromeric regions constitute natural AP brain developmental units shared by all vertebrates, each characterized by a distinctive *molecular* *profile* (a combination of active and inactive developmental genes—mostly transcription factors—which jointly control the activation at each distinct unit of particular cascades of downstream genes. Consequently, this entails differential sequential histogenetic phenomena all the way to adult fate. Individual genes may be shared in the profiles of adjacent or distant units, but each local combination is unique (sharing of some genes may lead to similarities in the final structure, as, e.g., presence of motoneurons as a local property). However, all these neuromeric histogenetic units soon become subdivided dorsoventrally (by parallel, orthogonally oriented DV molecular signaling, and consequent variations in the molecular profile) into a primary pattern of DV longitudinal zones, classically known as “*floor, basal, alar and roof plates*” (His, 1904). The resulting, subtly modified molecular profile of these zonal longitudinal domains within each neuromere diversifies the local histogenetic fates (e.g., types and number of neurons that can be produced). Some properties are shared along the whole length of these zones, that is, in all neuromeres (in some cases only in particular spans of such units). Both the neuromeres and their primary DV zones often register subsequently more advanced partial AP or DV regionalization. This generates (e.g., within the primary basal and alar plates) a number of smaller neuroepithelial subregions known as *microzones*, whose differential molecular profile becomes finally stable and homogeneous among an entire well-delimited neuroepithelial cell population. The microzones are also known as *progenitor areas* and have typologically quite specific neuronal derivatives, which may aggregate together at the local mantle layer, or disperse variously into neighboring or distant regions, mixing with other cell types. In the wall of the spinal cord myelomeres there appear in general five basal microzones and six alar ones; this number is roughly maintained along the hindbrain, with occasional variation in some of its neuromeres (Puelles, 2013); the final microzonal pattern is less well understood in the forebrain (but see Puelles et al., 2012a). It is well possible that microzonal alar and basal divisions basically continue showing a similar number in the forebrain, with changes mainly in their relative dimensions (larger DV dimension). In this respect, the behavior of the extraordinarily enlarged telencephalic field is exceptional, since it displays numerous further microzonal and areal subdivisions, particularly in the pallium; in contrast, the neural retina field also enlarges considerably in surface, but essentially remains a single microzone, unless we distinguish as such central, pericentral and peripheral retinal subregions. At the end of the regionalization process, the fully specified neuroepithelial microzones thus represent a definitive set of neural progenitor domains, which are each differentially specified molecularly in a way that confers to them quite distinct neural potencies and fates.

As a background for the present study, we need to give a brief introduction to forebrain neuromeric units. Neuromeres, in general, may be classified into three large tagmatic regions: 7 *prosomeres* in the recently expanded forebrain (the latter now includes the secondary prosencephalon, the diencephalon and the midbrain), 12 *rhombomeres* in the hindbrain, and over 30 *myelomeres* in the spinal cord (Figure 1A). These three initial tagmatic domains first divide into proneuromeric regions, which subsequently subdivide into the final neuromeric units. The forebrain is AP-regionalized into three proneuromeres called *secondary prosencephalon*, *diencephalon* and *mesencephalon* (Figure 1A; Puelles, 2013, 2018). The secondary prosencephalon (rostralmost forebrain component) will develop two hypothalamo-telencephalic prosomeres (hp1, hp2; Figure 1A), which will generate hypothalamic and telencephalic derivatives (the telencephalon is an expansive *alar* hypothalamic outgrowth, as are the eye cups and stalks). The diencephalon develops three diencephalic prosomeres (p1, p2, p3; Figure 1A). These units will be the source within the alar plate of the well-known pretectal (p1), thalamic (p2) and prethalamic (p3) regions. The midbrain represents the caudal-most forebrain region and contributes two midbrain prosomeres of unequal size (m1, large, and m2, small; Figure 1A). We do not need to detail the 12 neuromeric subdivisions which develop in the hindbrain (rhombomeres r0-r11; Figure 1A) or spinal cord (myelomeres; not shown; Puelles, 2013; Puelles and Rubenstein, 2015; Albuixech-Crespo et al., 2017).

As mentioned above, fate mapping studies in several vertebrates (teleosts, amphibia, birds and mammals), as well as longitudinal ontogenetic descriptive analysis of differential gene expression, have allowed to correlate at least partially the early transverse neuromeric and longitudinal zonal units and their respective ulterior microzonal subdivisions with the derived, anatomically characteristic, parts of the adult brain. The relevant conclusions on these fates have been abundantly corroborated with other approaches such as, e.g., experimental embryology and transgenic phenotypes (patterning analysis), chemoarchitecture, and genoarchitecture. This implies that it is possible to extrapolate early embryonic data on regionally discrete vascular invasion patterns with adult patterns of vascularization, using available fate maps.

Blood vessels do not yet invade the neural primordia at neural plate and early neural tube stages. The vascularization of the CNS begins shortly after the early stages of molecular regionalization of the tubular neuroepithelium take place. This process appears to be closely related with increased demands of oxygen and nutrients by neural progenitors when they initiate neurogenesis (Fish and Wythe, 2015; Tata et al., 2015). There are two distinct phases in CNS early vessel formation. During the first phase, known as *phase of* *external vascularization* (or *vasculogenesis*), angioblasts from the lateral plate and paraxial mesoderm produce endothelial cells that coalesce and differentiate into a primitive vascular network that covers superficially the entire neural tube; this network is identified as the *perineural vascular plexus* (PNVP; Figure 1B). This process occurs between E8.5 and E10 in the mouse and days 2–4 *in ovo* in the chicken; the human PNVP is observed at six gestation weeks (Marín-Padilla, 2012). During the following *phase* of *internal vascularization*, individual vessels sprouting from the PNVP perforate the piamater and penetrate the parenchyma of the brain tissue (*angiogenesis*). These initial *perforating vessels* seem to follow a straight radial course between the external limiting membrane and the ventricular surface (PV; Figure 1B). Once they are *inside* the ventricular zone, close to the ventricular lumen, they tend to produce circumferential branches at right angles (i.e., parallel to the ependym), which fuse with similar branches from other penetrating radial vessels, giving rise to a *periventricular vascular plexus* (PVVP; Figure 1B; Evans, 1909; Craigie, 1955; Stewart, 1955; Bär and Wolff, 1972; Bagnall et al., 1989; Couly et al., 1995; Kurz et al., 1996; Ruhrberg and Bautch, 2013; Fish and Wythe, 2015).

At later stages, after histogenetic growth of the mantle layer progresses, new radial vessels penetrate and additional collateral circumferential branches are produced within the mantle, which fuse or ramify as needed to cover the local vascular needs. Many of the added penetrating radial vessels remain restricted to given strata of the mantle layer. Marín-Padilla (2012) states that after 12 gestation weeks in human embryos, there is a constant distance of some 400 μm between each pair of penetrating vessels, from which it is deduced that a new PV is presumably intercalated wherever neural surface growth causes this spatial threshold to be surpassed. Indeed, the mean intervascular distance does not change between 12 gestation weeks and birth, with a hundredfold change in total brain weight (from 4 to 410 grams). Marín-Padilla (personal communication) thinks this threshold is due to a mean diffusion range of oxygen, which is efficient only within a radius of some 200 μm around the perforating vessel.

However, the precise temporospatial pattern obtained during brain vascularization is controversial, insofar as no attention has been given to such regional elements as proneuromeric regions and/or their neuromeric subdivisions, or to possible angiogenetic differences between the precociously differentiated basal plate and the more retarded, but more extensive alar plate. This analytic neglect obeys to the prominence during the relevant historic period of the columnar brain model, which considered transverse subdivisions unimportant (or inexistent). Early authors mapping vessel penetration in the forebrain and hindbrain regions (e.g., those cited above) generally considered this a sequential wave-like propagated process that starts in the caudal medullary rhombencephalon close to the spinal cord and then progressively extends rostralward and caudalward, until covering the whole brain. Any heterochronic vascular observation due to advanced vs. retarded neuromeres within the diverse brain regions was necessarily interpreted as an irrelevant variation within the simplistic columnar paradigm. Interestingly, an expanding general wave starting at the lower medulla was also the spatiotemporal pattern described in the same historic period for *precocious neurogenesis*. This view on wave-like neurogenesis was later corrected once it was discovered that paired rhombomeres (r2, r4, r6) develop in advance of unpaired ones (thus becoming the ones that carry the cranial nerve roots). This alternation generates subtle heterochronic aspects that had gone undetected before neuromeric models started to be contemplated (see, e.g., Puelles et al., 1987; Puelles, 2018). Marín-Padilla (2012) still described vascular invasion as starting at the caudal medullary rhombencephalon and progressing wave-like rostralwards through the rostral rhombencephalon, midbrain, and diencephalon, to finally reach the telencephalic region, thought to be located most “rostrally and dorsally.” Consciously or not, this description assumes the columnar model, which wrongly defines the telencephalon as the rostralmost forebrain portion. The prosomeric model instead visualizes the rostralmost forebrain as represented by the whole secondary prosencephalon (hypothalamus, eyes and telencephalon), where the telencephalon is conceived as a dorsal hypothalamic outgrowth (Figure 1A).

Other authors (Vasudevan et al., 2008) analyzing specifically telencephalic angiogenesis in mouse embryos observed precocious perforating vessels sprouting from the PNVP into the presumptive ganglionic eminences at E9.5, with subsequent “gradiental progress” of the invasion from subpallial into pallial regions (i.e., microzonal subdivisions of the telencephalic field). The telencephalic PVVP reportedly appears completed at E11 (Vasudevan et al., 2008). On the other hand, mouse hindbrain studies described the most precocious perforating vessels at E9.5 and earliest PVVP formation at E10.25 (Fantin et al., 2010). According to Daneman et al. (2009), sprouting of PVs from the PNVP begins uniformly at E10.5 in mouse. A shared stage of initial penetration at the telencephalon and hindbrain apparently weighs against the conventionally assumed overall caudorostral gradient.

The neuroepithelium is held to produce signals that stimulate external (PNVP) and internal (perforant vessels and PVVP) vascularization. The *vascular endothelial growth factor A* (VEGF A) produced by neural progenitors under hypoxic conditions is possibly the main stimulus for early neural vasculogenesis and angiogenesis. Apparently, this factor also seems the vehicle of positional information for heterochronic vessel formation (Hogan et al., 2004; Coultas et al., 2005; Santhosh and Huang, 2015). VEGF binds to tyrosine kinase receptors (VEGFR) present on the PNVP endothelial cells, as well as on the perforating vessels and their PVVP branches (Tata et al., 2015). VEGF-A/VEGFR2 (*Flk1*, *Kdr*) is the most important signaling pathway for early angiogenesis, and its genetic deletion is known to be lethal (Koch et al., 2011). The entrance of blood vessels into the brain is also strongly modulated by VEGF isoforms (Tata et al., 2015). In addition, canonical Wnt signaling from radial glia cells is another key element for vasculogenesis and angiogenesis in the neural tube. Wnt7a/7b ligands activate the canonical GSK/β-catenin pathway in endothelial cells, apparently aiding them significantly in their penetration (migratory) activity at early steps of vessel formation (Stenman et al., 2008; Daneman et al., 2009). Later in embryogenesis radial glia cells turn off the *Wnt* canonical pathway, thus contributing to vessel stabilization (Ma et al., 2013).

Regardless of evidence that arterial and venous vessels may show characteristic molecular differences from early developmental stages (e.g., *neuropilin* 1 and 2; Herzog et al., 2001), use of *Vegfr2* expression as a panendothelial vascular marker is convenient for the analysis of overall temporo-spatial patterns in early forebrain vascularization of mouse embryos. We compared at various early stages by *in situ* hybridization this vascular marker with some well-known regional markers of molecularly-defined neuroepithelial domains, consistently with our own earlier prosomeric studies (e.g., Dlx5, Pax3, Pax6, Shh, and Tcf7l2; Puelles and Rubenstein, 2003, 2015; Ferran et al., 2007, 2008, 2015a,b,c). We found that the PNVP is still incomplete at stage E8.5, but appears best developed next to the alar plate region of the forebrain. Some precocious perforating vessels (PVs) are seen from E8.5 onwards at various unrelated sites (heterotopy), leading subsequently also to independent incipient formation of the PVVP at specific neural domains. Vascular perforation thus follows in the space of the brain wall a heterochronic pattern that disagrees with any overall caudorostral or ventrodorsal gradients but is consistent with neuromeric and zonal brain wall subdivisions. We discuss whether these data, taken jointly with existing knowledge on general neural production of VEGF-A, are on the whole consistent with the existing theoretic notion that the heterochronic order of vascular invasion may reflect underlying *neurogenetic heterochrony* characteristic of differentially fated neural domains (e.g., predicting basal plate earlier than alar plate). The results seem partially contradictory with this interpretation, insofar as the early PNVP formation at alar levels coincides with a retarded local neurogenetic pattern, whereas neurogenesis advances precociously in an initially non-vascularized basal plate domain. We thus hypothesize that vascular penetration may obey different attracting mechanisms (signaling pathways) for PNVP and PVs formation, as well as for alar vs. basal brain territories. The expanded forebrain (including midbrain) may also follow different rules than the hindbrain and spinal cord. A partial causal connection of vascular penetration with local neurogenesis may obtain independently at some loci within these separate fields.

This analysis opens a new scenario in which to study the topology and local trajectory of major vascular entities relative to fate-mapped derivatives of the different developmental histogenetic units represented in the mature brain, naturally keeping in mind the accompanying anatomic deformations due to differential expansion/compression and morphogenesis of adjacent developmental units. This novel sort of analysis is attempted here in a tentative way, using the more detailed adult human data from the literature. The resulting prosomeric vascular map shows remarkably salient features. We envisage that one possible end result may be a complementary developmental nomenclature of brain vessels. In principle, this might be useful for some clinical applications (e.g., in interventional radiological analysis of arterio-venous malformations, or in selective chirurgical obturation of some vascular pedicles).

## Materials and Methods

### Mouse Embryos

All experimental procedures were conducted according to the legislation from the European Community (86/609/EEC) and Spanish Government (Royal Decree, 1201/2005; Law 32/2007). All mouse experiments were approved by the ethical committee from the University of Murcia. *Swiss albino* mouse embryos staged according to Theiler criteria (TS; Theiler, 1989) were collected at different embryonic days (E) after fertilization (see text and Figures). At least 10 embryos were analyzed at each selected stage and three or four series of sections were obtained from each brain to analyze different markers (see below). Some additional expression patterns of *Vegfr2*, *Eng* and *Ctgf* were obtained from *in situ* hybridization images downloaded from the Allen Developing Mouse Brain Atlas.

### Tissue Processing

All the experimental procedures related with extraction and processing of brain samples in embryos were performed as previously described (Ferran et al., 2015a). Brains were fixed in phosphate-buffered 4% paraformaldehyde (0.1 M PB; pH 7.4) at 4°C for 24 h. Afterward, embryonic brains were transferred to 30% sucrose in 0.1 M PBS (phosphate-buffered saline solution) and then embedded in 15% gelatin/20% sucrose. Serial 20 μm-thick sections were obtained using a cryostat (Leica CM3500 S), collected as parallel series on SuperFrost Plus slides (Menzel-Gläser, Braunschweig, Germany), and stored at −20°C.

### RT-PCR

*Pax3, Pax6, Tcf7l2* and *Vegfr2* cDNA fragments were obtained by reverse transcription (RT). RNA was extracted with Trizol reagent (Invitrogen, Carlsbad, CA, USA) from fresh dissected brains of *Mus musculus* embryos. The RNA was treated with DNase I (Invitrogen, Carlsbad, CA, USA). RNA samples were then retro-transcribed into single-stranded cDNA with Superscript III reverse transcriptase and oligo dT anchored primers (Invitrogen, Carlsbad, CA, USA, SuperScript First-Strand Synthesis System for RT-PCR). The cDNA was used as a template for PCR with *Taq* polymerase (Promega, Madison, WI, USA) and specific primers. The PCR products were cloned into pGEM-T Easy Vectors (Promega, Cat. A1360) and sequenced (SAI, University of Murcia, Murcia, Spain). Primers:

- MPax3F: 5′ TACCAGCCCACGTCTATTC 3′
- MPax3R: 5′ AGGTCATGCTGGGACAATTC 3′
- MPax6F: 5′ GGCCAGCAACACTCCTAGTC 3′
- MPax6R: 5′ TGTGTGTTGTCCCAGGTTCA 3′
- MTcf7l2F: 5′ AAAATGCCGCAGCTGAACG 3′
- MTcf7l2R: 5′CCATATGGGGAGGGAACC 3′
- MVegfr2F: 5′ AGCGTTGTACAAATGTGAAG 3′
- MVegfr2R: 5′ CTGGCATCATAAGGCAAGCG 3′

### *In situ* Hybridization

All the steps followed during the entire procedure are detailed in Ferran et al. (2015a,b). Sense and antisense digoxigenin-UTP-labeled riboprobes for mouse *Dlx5*, *Pax3, Pax6, Shh*, *Tcf7l2* and *Vegfr2* were synthesized according the manufacturer’s suggestions (Roche Diagnostics S.L., Applied Science, Barcelona, Spain) and using specific polymerases (Fermentas, Madrid, Spain). Probe sequence information is provided in Table 1. Hybridizations were carried out overnight at 72°C. RNA-labeled probes were detected by an alkaline phosphatase-coupled anti-digoxigenin antibody (diluted 1:3.500; Roche Diagnostics, Manheim, Germany), and the compound nitroblue tetrazolium/5-bromo-4-chloro-3-indolyl phosphate (NBT/BCIP; Roche Diagnostics, Manheim, Germany) was used as a chromogenic substrate for the alkaline phosphatase reaction.

**Table 1:** Probes.

Gene symbol	NCBI accession no.	Size (bp)	Positions	Publication/Laboratory
*Dlx5*	NM_010056.2	1,180	106–1,285	Morales-Delgado et al. (2011)
*Pax3*	NM_008781.4	953	1,321–2,273	Present results
*Pax6*	NM_001244198.2	928	1,158–2,085	Present results
*Shh*	NM_009170.2	643	442–1,084	McMahon A. lab
*Tcf7l2*	NM_001142918.1	826	530–1,355	Present results
*Vegfr2*	NM_01612.2	900	1,829–2,728	Present results

### Imaging

Digital images were obtained with a ScanScope CS digital slide scanner (Aperio Technologies, Vista, CA, USA). Contrast and focus were adjusted by applying Adobe Photoshop CS3 software (Adobe Systems Inc., San Jose, CA, USA).

## Results

During the determination of artery or vein identity, several molecules are involved in the differential specification of their endothelial cells. According to a number of studies, genes involved in the promotion of an arterial identity include *EphrinB2a, Shh, Ihh, Notch1/4, Jag1/2, Dll4*, and *Np1*; a venous identity obeys instead to the activity of *COUP-TFII, Np2, EphB4* and *Vegfr3 (Flt4)*. However, most of these determinants are not exclusive arterial or vein markers (they appear active also in other developing systems), and not all of them are expressed at early stages in the whole arterial or venous network of the brain (Swift and Weinstein, 2009; Fish and Wythe, 2015). Having in mind the difficulty to find selective markers for the whole brain arterial or venous network from early stages of development onwards, we opted for one of the well-known panendothelial markers (*Vegfr*1 or *rFlt1*, *Vegfr2* or *Flk1/Kdr*, *Cdh5* or *Eng*; Swift and Weinstein, 2009). We elected *Vegfr2* (*Flk1/Kdr*) for our study because it is highly expressed from the beginning of vascularization of the CNS and during early stages of development in the entire vascular network of the brain.

### The Perineural Vascular Plexus (PNVP) and First Perforant Vessels (PV) at E8.5 Stage

**Figure 2:**
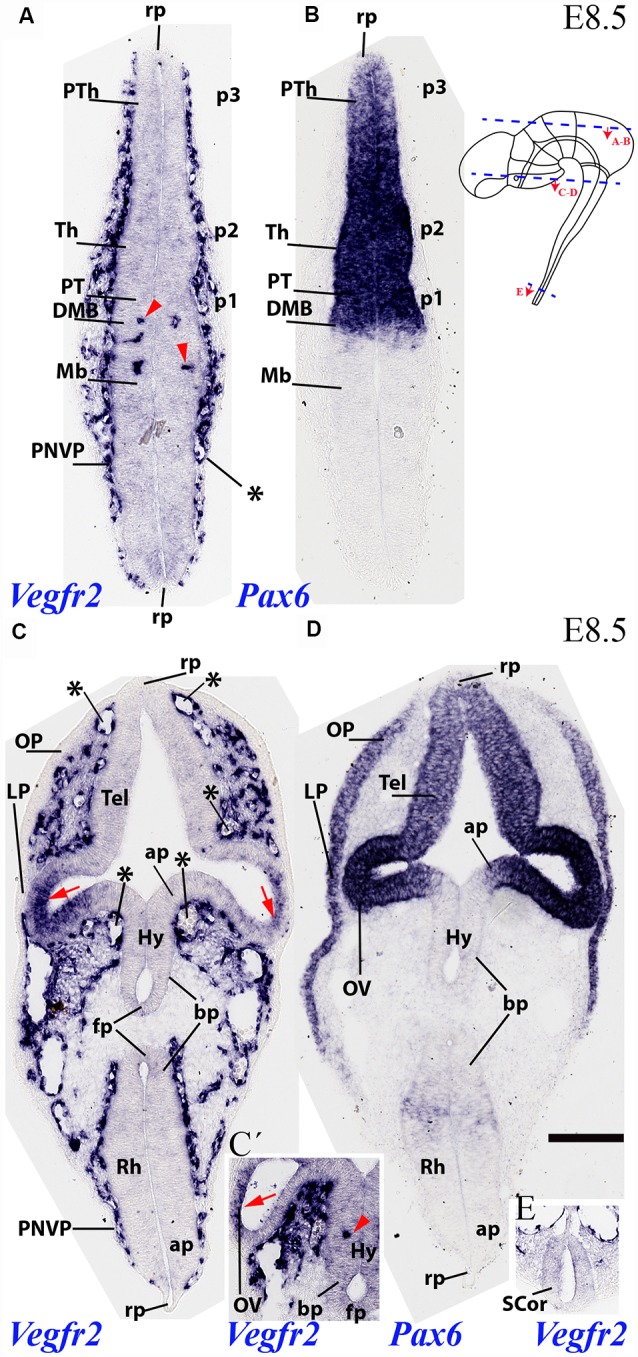
Two horizontal sections through mouse secondary prosencephalon, diencephalon and mesencephalon at E8.5 showing varying degrees of external vascularization by the PNVP (labeled with endothelium-selective Vegfr2 riboprobe; **A**,**C**), compared in consecutive sections with Pax6, an alar plate marker for the diencephalic neuromeres p1-p3 **(B)** and the secondary prosencephalon (telencephalon and optic vesicle in **D**). Inset to **(B)**: semi-schematic representation of the plane of section of the horizontal sections **(A,B)** and **(C,D)**. Note the PNVP-nude roof plate area rostrally and caudally (rp; **A**). The earliest Vegfr2-positive perforant vessels are marked with red arrowheads in **(A)** and inset (**C’**; detail of alar hypothalamus, Hy). The red arrows in **(C)** point to areas of non-vascular Vegfr2 expression at the neural retina primordia. Note also in **(C)** the remarkable lack of PNVP at the forebrain and hindbrain floorplate (FP) and neighboring basal plate (bp). The median and paramedian perichordal mesoderm also lacks vascularization, in contrast with lateral mesoderm areas next to the neural alar plate (unlabeled). Inset **(E)** shows the very retarded state of vascularization at the spinal cord (SCor). Asterisks: developing venous seins. Scale bar: 150 μm.

The analysis of *Vegfr2* expression at E8.5 shows that external vascularization is highly developed, but a dense perineural vascular plexus (PNVP) does not yet cover the entire brain surface, relating preferentially to alar portions of the neural tube. A horizontal section through dorsal alar territories of diencephalon (*Pax6*-positive) and midbrain (*Pax6*-negative) shows abundant PNVP next to the alar pial surface, but no PNVP at the respective roof plate sites (rp; Figures 2A,B; section level marked in the inset drawing). We can see also that there appear incipient perforating vessels inside the caudal-most diencephalon and rostral alar midbrain (red arrowheads; Mb; Figures 2A,B; note none more caudally in the midbrain). The DMB tag marks the di-mesencephalic boundary, which is underlined molecularly by selective diencephalic expression of *Pax6* as a progressive site for vascular penetration (compare Figures 2A,B), irrespective that the corresponding roof, basal and floor plates are devoid of PNVP.

Another section level from the same E8.5 specimen intersected transversally the secondary prosencephalon and obliquely the hindbrain (level in the inset drawing), showing in both cases the vascular pattern of both alar and basal plates, as well as the roof and floor plates (Figures 2C,D). *Pax6* mRNA is present at this stage in an upper part of the alar plate of the secondary prosencephalon, including the eye stalk and eye vesicle (strong *Pax6* expression) and the neighboring telencephalic stalk and pallium (weaker expression). The *Vegfr2* signal shows some large or medium size vessels (probably venous sinuses) associated to the pallial telencephalic surface, but there is no continuous PNVP yet at this site (asterisks mark these large vessels; Tel; Figure 2C); moreover, the telencephalic roof plate is wholly devoid of PNVP (rp; Figure 2C). The eye stalk area is already surrounded by a thick PNVP, but not so the peripheral part of the optic vesicle (OV) whose prospective neural retina field shows itself marked neuroepithelial *Vegfr2* expression, possibly responding to signals emanating from the lens placode (OV; red arrows; LP; Figures 2C,D).

There appear at this level three particularly large venous blood vessels, in a dorsoventral pattern (next to roof plate, and above and under the eye stalk; asterisks; Figure 2C). The associated hypothalamic PNVP seems to cover exclusively the *Pax6*-negative/*Dlx*-positive alar (*Dlx* pattern not shown) hypothalamus (ap), contrasting with a nude hypothalamic basal plate (bp) and an associated clearcut lineal boundary between ventral avascular and dorsal vascularized paramedian mesoderm. The hypothalamic floor plate (fp) is also nude of PNVP (Hy; ap; bp; fp; Figure 2C). The inset Figure 2C′ shows a more intensely reacted detail of an adjacent section, showing an isolated perforating vessel observed within the alar hypothalamus at this stage (Hy; red arrowhead; the red arrow points to *Vegfr2*-positive prospective neural retina, as in Figure 2C). In contrast with these precocious forebrain areas, the hindbrain (Rh; fp; bp; ap; rp; Figure 2C) and spinal cord (SC or; Figure 2E) are still devoid of PVs, and the spinal cord also lacks a PNVP.

### PNVP, Penetrating Vessels (PV) and First Periventricular Vascular Plexus (PVVP) at E9.5

**Figure 3:**
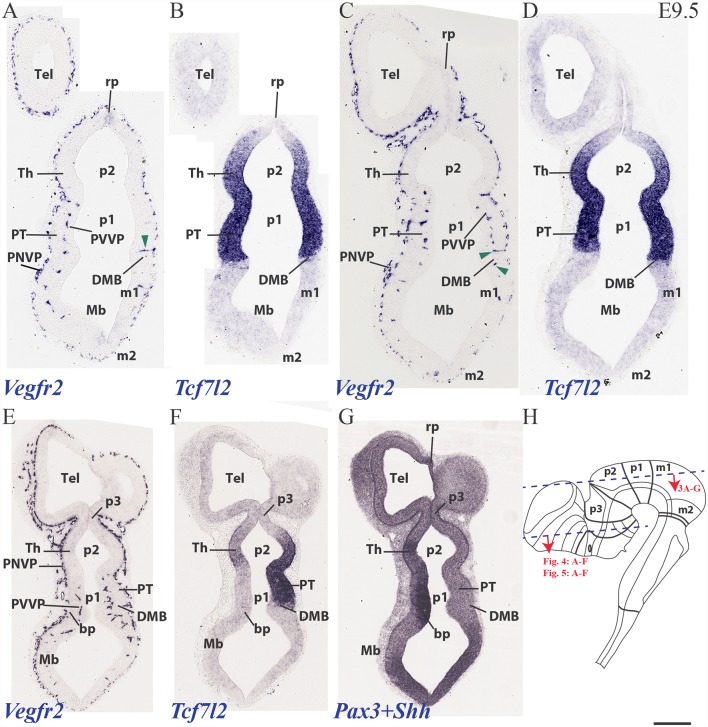
Horizontal sections through a mouse telencephalon, diencephalon and mesencephalon at E9.5 showing the vascularization by the PNVP and early perforant and periventricular (PVVP) vessels (Vegfr2; **A,C,E**), compared in consecutive sections with markers of diencephalic alar plate (Tcf7l2 and Pax3; **B,D,F,G**) and basal plate (Shh; **G**). See description in the text. Green arrowheads: perforant vessels. **(H)** Semi-schematic representation of the plane of section corresponding to the horizontal sections shown in Figures 3–5. Scale bar: 250 μm.

**Figure 4:**
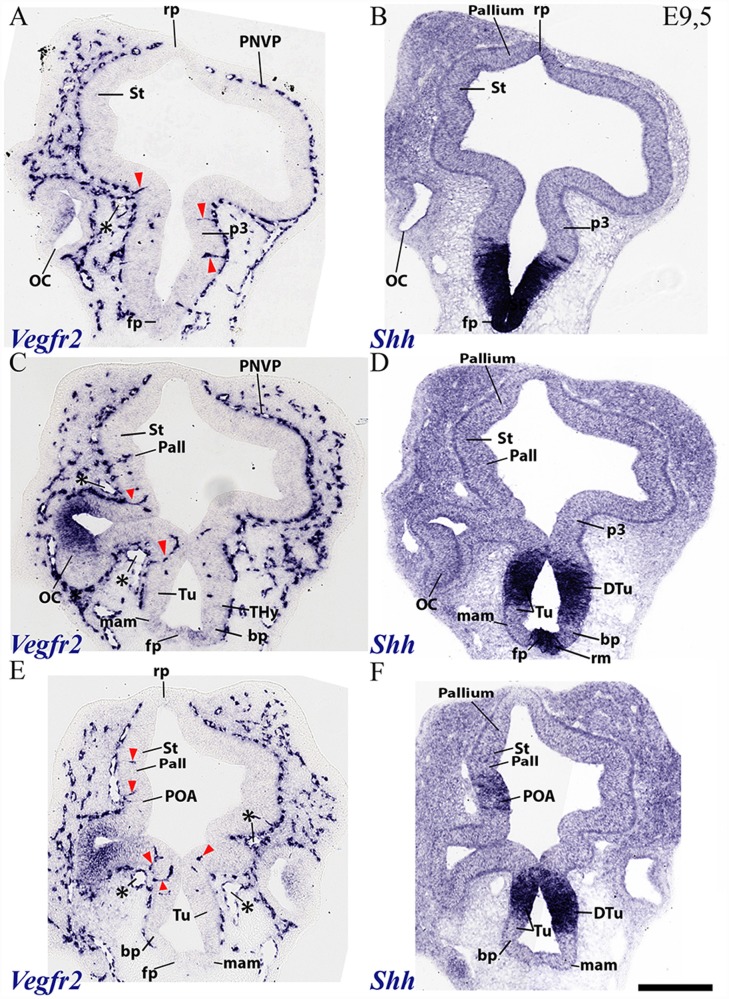
Horizontal sections through a mouse secondary prosencephalon and diencephalon at E9.5 showing PNVP vascularization and first perforant vessels (Vegfr2; **A,C,E**), compared in consecutive sections with a marker of hypothalamic and diencephalic basal plate (Shh; **B,D,F**). See schematic representation of plane of section in Figure 3H. See description in the text. Red arrow heads: perforant vessels. Asterisks: presumed venous sinuses. Scale bar: 250 μm.

**Figure 5:**
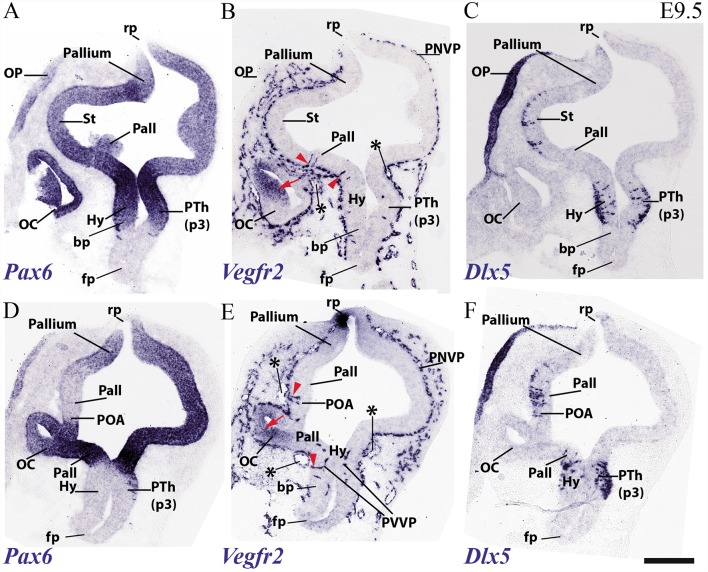
Transversal sections through a mouse secondary prosencephalon and diencephalon at E9.5 showing PNVP vascularization and first perforant vessels (Vegfr2; **B,E**), compared in consecutive sections with markers of telencephalic pallium and striatum (Pax6; **A,D**) and telencephalic subpallium and part of alar hypothalamus (Dlx5; **C,F**). Red arrowheads: perforant vessels, red arrow: non-vascular expression of Vegfr2 at the retinal primordium of the optic vesicle. Asterisks: presumed venous sinuses. See schematic representation of plane of section in Figure 3H. Scale bar: 200 μm.

At E9.5, the neuromeres start to grow in surface, limited by their non-growing transverse interneuromeric boundaries, as best visualized in horizontal and sagittal sections. The major DV subdomains become molecularly identifiable. We accordingly compared at this stage *Vegfr2-*expressing vessels with *Dlx5*, *Pax3*, *Pax6*, *Shh* and *Tcf7l2* mRNA areal neuroepithelial or mantle (neuronal) expression in consecutive horizontal sections (Figure 3). The same section plane (illustrated in Figure 3H) cuts transversally the secondary prosencephalon (Figures 4, 5), due to the cephalic flexure. With the cited genoarchitectonic markers it is possible to recognize subpallial vs. pallial telencephalic subdomains, and some alar and basal hypothalamic and diencephalic domains. The PNVP covers at E9.5 practically the entire alar and basal plates of the prosencephalon, with the exception of the rostralmost basal plate at the median tuberal acroterminal region and possibly a paramedian basal band next to the floor plate, where a PNVP is still absent. Some scattered vessels appear over the midbrain and hindbrain roof plate (Figures 3–5).

The PNVP covering alar diencephalon and midbrain seems complete but somewhat stretched out (less thick than at E8.5), possibly due to the intervening surface expansion of these brain units. The horizontal sections through the forebrain shown in Figures 3A–H, where *Tcfl2* expression labels selectively the p1 and p2 alar plate domains (pretectum or PT in p1, thalamus or Th in p2; Figures 3B,D,F), show an increasing number of PVs contributing to an incipient PVVP formation across the three dorsoventral section levels shown (Figures 3A,C,E). This pattern is nevertheless restricted to PT and rostral midbrain (Mb). A detailed analysis of the radial course of the pretectal perforant vessels (PVs) strongly suggests that these vessels never cross interprosomeric boundaries [e.g., green arrowhead pointing to a pretectal PV entering just in front of the di-mesencephalic boundary (DMB) Figure 3C].The incipient PVVP seems clearly most advanced at the ventralmost level (PVVP; Figure 3E), in a section that lies close to the alar-basal boundary (note transition from alar *Tcf7l2* expression in Figure 3F, right side, into basal *Shh* expression in Figure 3G, left side). The alar midbrain shows on the whole fewer PVs than the PT, and they are now markedly scattered caudalwards, possibly due to differential interstitial growth (Mb; m1; Figures 3A,C,E); no significant midbrain alar PVVP is apparent, except close to the basal plate (Mb; bp; Figure 3E). In contrast, the thalamus in p2 (p2; Th; PNVP; Figures 3A,C,E) and the prethalamus in p3 (p3; Figures 3E,F) appear covered by a full alar PNVP since E8.5, but show no PVs yet at E9.5. The earliest prethalamic PVs are found at the rostral end of this neuromeric domain (red arrowheads; p3; Figures 4A,C, 5B).

**Figure 6:**
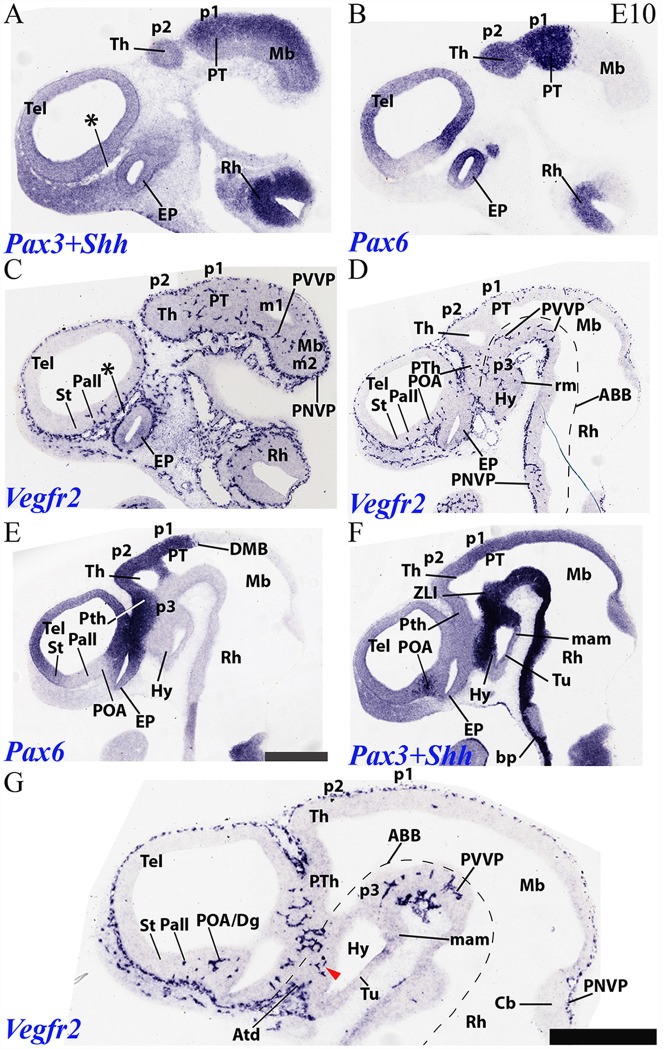
Sagittal sections through a mouse forebrain and hindbrain at E10 (in lateral to medial order) showing PNVP vascularization, perforant vessels and PVVP formation (Vegfr2; **C,D,G**), compared in consecutive sections with markers of alar forebrain (Pax6; **B,E**) and alar and basal forebrain/hindbrain (Pax3+Shh; **A,F**). Red arrow heads: earliest PVs at the dorsal part of the hypothalamic basal plate; note more advanced local alar plate. Asterisks: presumed venous sinuses. See further description in the text. Scale bar: 400 μm.

The dorsocaudal parts of the telencephalic vesicle sectioned in the Figure 3 series (see drawing in Figure 3H) are the most immature ones in terms of proliferation and neurogenesis. There is here a rather uniform PNVP cover, possibly weaker next to the median roof plate, but no PVs are present (Tel; Figures 3A,C,E). In contrast, the telencephalic sections illustrated in Figures 4, 5 are topological transverse sections through the secondary prosencephalon (see drawing in Figure 3H; in both cases, the levels proceed caudorostrally). Figure 4 compares *Vegfr2* with the floor and basal marker *Shh* (noting there is a tuberal and mamillary basal patch in the hypothalamus that secondarily downregulates its primary *Shh* expression; compare *Shh*-negative basal plate areas in Figures 4D,E with the sagittal section at E10 in Figure 6F). The upper boundary of the *Shh* signal marks the alar-basal limit throughout (Figures 4B,D,F, 6F).

The overall cover of PNVP at the section levels shown in Figure 4 has expanded more fully towards the roof plate, and also extends now more ventralwards in the hypothalamus, where PNVP and PVs are found now both in its alar and upper basal regions, though respecting still the ventralmost region next to the floor plate, where the mamillary pouch lies (red arrowheads; fp; bp; Tu; mam; rm; Figures 4A–F). The alar hypothalamic areas around the optic stalk show the best developed PVs (red arrowheads; Figures 4A,C,E). The optic stalk and prospective pigmented retina are provided already by a PNVP, but are devoid of PVs, while the neural retina itself continues to express *Vegfr2* (Figures 4A,C,E, 5B,E). We still see large venous blood vessels below and above the optic stalks (asterisks; Figures 4C,E). The hypothalamic floor plate and ventral part of the basal plate continue nude of PNVP, in parallel with its neighboring mesoderm.

As regards the telencephalon, we observed at E9.5 the earliest PVs within the subpallium, particularly at its incipiently defined preoptic area subdomain, recognized by its characteristic selective expression of *Shh* (within the alar plate; red arrowheads; POA; Figure 4F), but possibly also within *Shh*-negative pallidum (red arrowheads; Pall; Figure 4C). The striatum seems still devoid of PVs (St; Figures 4A,C,E).

**Figure 7:**
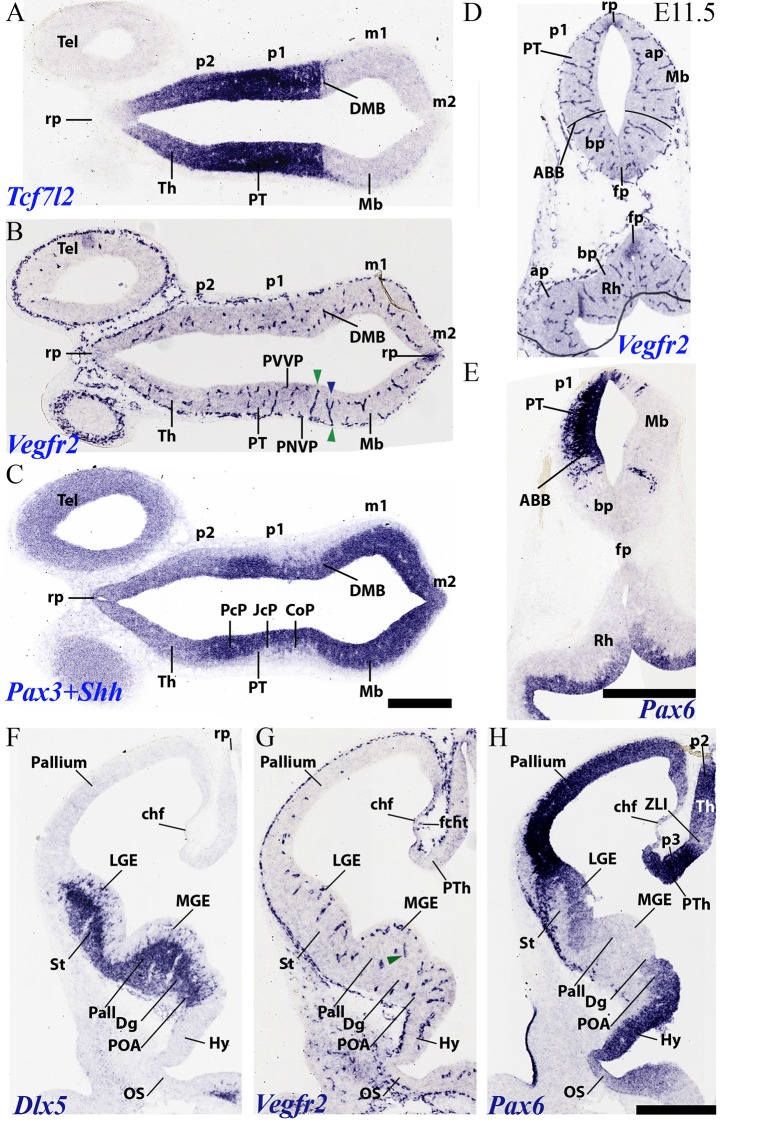
Horizontal **(A–C)** and transversal **(D–H)** sections through embryonic mouse alar forebrain at E11.5 showing PNVP vascularization, perforant vessels and PVVP formation (Vegfr2; **B,D,G**), compared in consecutive sections with various markers of the alar forebrain (Tcf7l2: **A**; Pax3+Shh: **C**; Pax6: **E,H**, and Dlx5: **F**). **(A–C)** Horizontal sections displaying diencephalic neuromeres p2 (Th) and p1 (PT) and midbrain (Mb), jointly with dorsal part of telencephalic vesicle (Tel), in order to observe segmental differences in degree of alar vascularization. **(D,E)** Consecutive transversal sections passing through the midbrain-diencephalic border (left side = PT; right side = Mb), and showing also a hindbrain cross-section underneath (Rh). The alar-basal boundary is indicated (ABB), as delineated by Pax6 alar signal in **(E)**. Green arrowheads: perforant vessels restricted radially to a specific neural histogenetic domain; blue arrowhead: a perforant vessel ramifies into PVVP within Mb, but does not invade adjacent PT. **(F–H)** Three consecutive cross-sections through the secondary prosencephalon (hypothalamus plus telencephalon), midways through the telencephalic vesicle, illustrating vascularization patterns (Vegfr2; **G**) in the subpallium (medial and lateral ganglionic eminences, MGE, LGE; marked by Dlx5 expression in **F**) and the pallium (marked by Pax6 expression in **H**; Pax6 signal also appears in the preoptic area, POA). See description in the text. Scale bars: 400 μm.

Figure 5 shows similar section levels as Figure 4, but it compares *Vegfr2* (Figures 5B,E) with *Pax6*, characteristic of the alar plate in the telencephalic pallium and diencephalon (Figures 5A,D, 6B,E) and *Dlx5* expression, present in the subpallium (Figure 7F) and the alar prethalamus (PTh; Figures 5C,F). The telencephalic subpallium shows weak *Pax6* signal at its prospective striatal subdomain but is *Pax6*-negative in its pallidal, diagonal and preoptic subdomains (Figure 7H; check also Puelles et al., 2000, 2013, 2016). As seen before in Figure 4, many PVs can be observed in subpallial and hypothalamic alar and upper basal domains, but PVs are still absent in the striatum as well as in pallial telencephalic regions (red arrowheads; Figures 5B,E). The subpallial region displays the largest number of PVs in the preoptic domain and fewer of them in the diagonal and pallidal neighboring domains. A comparison of *Vegfr2*, *Pax6* and *Dlx5* expression indicates that the hypothalamic PVs are localized at the E9.5 stage either at the dorsal tuberal area (upper basal plate) or at the subparaventricular/paraventricular areas (alar plate). Such PVs are still absent in the most basal (mamillary and perimamillary) domains next to the floor (mam; Figures 4C,E, 5B,E).

### PNVP, PVs and PVVP Vascular Pattern in the Prosencephalon of Mice at E10

We compared in an E10 sagittal section series *Vegfr2* signal (Figures 6C,D,G) with *Pax3* (a marker of midbrain and pretectal alar plate; Figure 6A), *Pax6* (marker of diencephalic and secondary prosencephalic alar plate, with exception of the *Dlx*-positive ventral subdomain of the hypothalamic alar plate; Figures 6B,E; Puelles et al., 2012a) and *Shh* (a floor and basal plate marker in the whole forebrain, except in a tubero-mamillary band within basal hypothalamus; *Shh* only labels floor plate in the hindbrain; Figure 6F). The series proceeds lateromedially. Lateral sections in Figures 6A–D first pass tangentially through the lateral alar wall of p1 and p2, plus the midbrain, and subsequent sections finally show the corresponding ventricular cavities. It can be observed that Th in p2 continues largely devoid of PVs, whereas PT in p1 displays them regularly, as well as the neighboring midbrain. The alar prethalamus also shows now a significant number of PVs (p3; PTh; Figures 6D,G), more or less in continuity with those in the alar hypothalamus (Hy; Figure 6G), and starts to build a local PVVP. The alar thalamic p2 field thus represents a non-invaded discontinuity (retarded heterochrony) within the central neuromeric unit of the diencephalon. As the sections approach the alar-basal boundary found underneath these alar regions (Figures 6E,F), we observe already in Figure 6D a significant number of PVs disposed uniformly along the Mb, PT, Th and PTh basal plate (tegmentum), even starting to form a PVVP. This basal PV pattern is reproduced less markedly in the hypothalamus (e.g., within the *Shh*-positive retromamillary area; Hy; Figure 6D, and the similarly *Shh*-positive dorsal tuberal area; red arrowhead; Hy; Figure 6G). Some PVs are found as well at the acroterminal (rostralmost) basal tuberal domain (Atd; Figure 6G).

*Pax6* and *Shh* labeling are useful to demarcate the different St, Pall, Dg and POA subdomains of the telencephalic subpallium (Figures 6E,F). This allowed us to corroborate at E10 our impression gained on E9.5 material that PVs are still selectively absent from the developmentally more retarded striatal subdomain, some PVs are present in the pallidum, and the largest number of PVs characterizes the diagonal and preoptic areas (St, Pall, Dg, POA; Figures 6C,D,G). No PVs are observed at the *Pax6*-positive pallial region. Note as well in Figure 6G that the cerebellar plate (Cb) shows a distinct PNVP, but no PVs, as occurs as well at the neighboring caudal midbrain.

### PNVP, PVs and PVVP Vascular Pattern in the Prosencephalon of Mice at E11.5

During this stage further neuromeric and telencephalic growth occurs, and the molecular diversity is increased by new inner partitions; moreover, the mantle layer increases considerably in thickness, but without reaching a final status yet (Figures 7A–H). This increases the radial complexity of the neural wall with particularities at each developmental unit. Axonal navigation has started as well, though identifiable fiber strata may be detected only at few places (e.g., the posterior commissure in Figure 7C). In the secondary prosencephalon, a notable change is represented by a large increase in thickness of the whole subpallial region, where a lateral intraventricular bulge known as the lateral ganglionic eminence (striatal domain), and a smaller medial intraventricular bulge defined as the medial ganglionic eminence (pallidal plus diagonal domains) are observed, next to the non-evaginated preoptic area (LGE, MGE, POA; Figures 7F–H). Hypothalamic dorsoventral microzonal subdivisions, and pretectal anteroposterior partitions become molecularly defined at E11.5.

At around this stage, the outer limiting membrane of the entire neural tube is covered by the PNVP; this includes hypothalamic basal acroterminal domains, as well as the previously uncovered floor and roof plates (Figures 6G, 7B,D,G).

The perforant vessels (PVs) in the alar diencephalon and midbrain are still most abundant, and are particularly visible at the pretectum (PT; p1; Figures 7A–C), where an anteroposterior alar regionalization into precommissural, juxtacommissural and commissural subdomains can be appreciated and distinguished molecularly (PcP, JcP, CoP; Figure 7C; Ferran et al., 2008). The CoP coincides with the aggregated transversally coursing fibers of the posterior commissure. The alar midbrain shows less PVs than the pretectum, but already displays a PVVP that reaches the *Vegfr2*-positive roof plate (Figures 7B,D; compare Figure 7E, a section roughly across the DMB, and showing as well the *Pax6*- expression limit at the alar-basal border; ABB). On the other hand, the alar thalamus domain shows now already an incipient PVVP, but PVs are rarely found (p2; Th; Figures 7B,G). This raises the possibility that this thalamic PVVP is largely an extension of the PVVP from the underlying p2 basal plate, rather than an independently formed alar one (see “Discussion” section below in connection with singular basal penetrating thalamic arteries). A characteristic basal plate pattern is observed in Figure 7D, which displays the Mb on the right side and the PT on the left side; the basal PVVP seems less developed than the alar PVVP. The midbrain and hindbrain floor plate expresses weakly *Vegfr2* (fp; Figure 7D), as does the midbrain and diencephalic roof plate (rp; Figures 7B,D).

The alar hypothalamus near the optic stalk shows PVs and an incipient PVVP (Hy; Figure 7G; not so the optic stalk itself, restricted to a PNVP). Proceeding from alar hypothalamus into subpallial telencephalon (the cited sizeable ganglionic eminences), we still observe a step-like change in the number of PVs across the POA, Dg, Pall and St subdomains. The striatal primordium now displays for the first time PVs and incipient PVVP (Figure 7G; compare limits in Figures 7F,H). Moreover, we also first see at E11.5 some PVs and an incipient PVVP at the pallial region adjoining the subpallium; the density of pallial vessels decreases gradientally towards the convexity of the hemisphere. The pallial area lying immediately next to the striatum is the ventral pallium, where the olfactory cortex is produced. This is followed by the claustro-insular complex, or lateral pallium, the neocortical primordium or dorsal pallium, the cingulate mesocortex and the hippocampal allocortex, or medial pallium, which would map on the medial wall of the hemisphere (Puelles et al., 2000, 2019; Puelles, 2014; Watson and Puelles, 2017). This medial wall also displays the thinner neuroepithelial tela of the chorioidal fissure (chf; Figures 7F–H), which interconnects the prospective hippocampal fimbrial taenia with a prethalamic taenia at the roof plate end of the prethalamic eminence (PTh; Figures 7G,H). The invasion of the future chorioidal plexus of the lateral ventricle through the chorioidal fissure has not yet begun at E11.5. In fact, there is only a tenuous PNVP at the outer or pial surface of the fissural chorioidal tela (fcht; Figure 7G).

### Topologic Positioning of Major Brain Vessels on the Prosomeric Model

The external vascularization by the perineural vascular plexus (PNVP) covers during early development the entire neural tube and will derive in the adult in a complex extracerebral compartment. This compartment is represented in adult animals by an external venous system (outer dural), a middle compartment of main arterial and venous vessels (arachnoidal layer), and an inner compartment represented by a pial anastomotic plexus. The blood supplied by the main arterial vessels reaches the arachnoidal layer, from where smaller branches connect variously with the capillary plexus covering the outer limiting membrane. Terminal vessels from this plexus penetrate the neural tissue and connect therein with capillaries (Marín-Padilla, 1987, 2012; Scremin and Holschneider, 2012; Scremin, 2015). Perforant vessels (PVs) sprout progressively from the PNVP, intercalating apparently at a standard mean distance of 400 μm (Marín-Padilla, 2012) as they penetrate the brain parenchyma along a more or less radial path that initially reaches the ventricular zone of the neuroepithelium, where final circumferential branches are given to build the PVVP (Figure 1B). At later stages, other lateral branches sprout from the PVs at several levels through the mantle layer. Numerous accounts and mappings exist about the main brain vascularization fields that correspond to branches of the vertebral, basilar, and internal carotid arteries.

While these facts are well known, our results on differential positional timing of PV entrance in relation to molecular compartments of the brain wall led us to become interested in an issue that apparently has never been considered before, namely the question whether the arachnoid vessels course and produce secondary branches in a specific topologic relationship with the brain’s subdivisions according to the prosomeric model (these are understood as *natural* developmental units of the brain, as opposed to other sorts of arbitrarily defined anatomic partitions; Nieuwenhuys and Puelles, 2016). Previous impulse towards exploring this issue came from the reported experience of interventional neuroradiologists with arteriovenous malformations; this pathology apparently often reveals peculiar positional restrictions (boundaries) of the abnormal vessels, which have been conjectured by Valavanis (2003) to be associated to molecular compartments of the brain wall. Assuming that the position of the main forebrain arterial branches is relatively well conserved in mammals and even in tetrapods (see however about rodent variations in Scremin, 2015), we opted in our analysis for the best known human arterial pattern.

**Figure 8:**
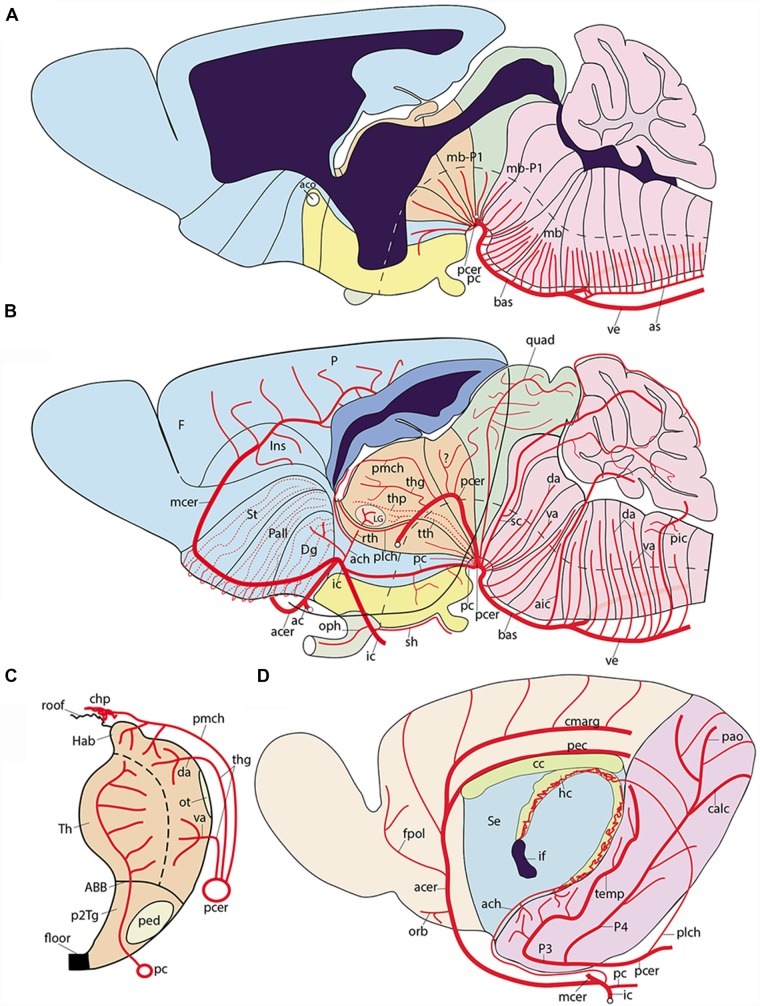
Semi-realistic schemata based on the updated prosomeric model shown in Figure 1A, illustrating the forebrain and hindbrain regions **(A,B)**, a thalamus cross-section **(C)**, and the isolated telencephalon **(D)**, cut in various ways to visualize vascular patterns relative to brain histogenetic units contemplated in the model (transversal neuromeres and dorsoventral alar/basal longitudinal zones). Note the color code of the different brain regions in **(A,B)** coincides with that in Figure 1A. **(A)** Schematic paramedian sagittal section (ventricular cavity in black), showing the origins, penetration sites and intraneural topologically transverse course of a continuous set of medial vessels serving the paramedian basal plate all the way into the hypothalamus; these fan out in the forebrain due to the axial incurvation at the cephalic flexure (note similarly bent alar-basal boundary drawn in as a longitudinal black dash line). The basal plate arteries sprout sequentially from the anterior spinal and vertebral arteries (as, ve), the basilar artery (bas), the stem of the posterior cerebral artery (pcer), and the posterior communicating artery (pc); this last artery is not shown, since it lies lateral to this nearly median plane of section). **(B)** Schema aiming to depict typical courses of arteries serving the alar plate domains of the brain (alar-basal boundary again as bent longitudinal black dash line). The alar plate vessels mainly derive from the ve, bas, pcer, mcer and pc arteries. In the hindbrain they first circumvent the basal plate domain with an initial ventrodorsal course in the subarachnoidal space, adapted topologically to the diverse neuromeric regions, and then they penetrate either ventral or dorsal parts of the corresponding alar plate sector (va, da); special cases are represented by the postero-inferior, postero-superior and superior cerebellar arteries, which produce va and da branches as well as chorioidal branches for their neuromere, and then jump into the overlying cerebellum. At the midbrain, the quadrigeminal artery behaves somewhat like a va + da artery, but the dorsalmost part of the colliculi are served by a hyperdorsal supracollicular network of da-like vessels (not shown). The diencephalon shows a contrasting pattern, insofar as alar arteries arise either as perforating arteries (from the pcer P1 segment, or the posterior communicating artery, pc; pcer; see cross-section in **C**), which first penetrate the basal plate and then continue internally dorsalward until reaching periventricular alar centers, or as dorsally coursing va, da or chorioidal branches of the posterior cerebral artery, which follows a longitudinal topologically rostralward course along the diencephalic ventral alar plate domain (pcer; its P2 segment), before it bends lateralwards into the posterior telencephalic cortex (P3 and P4 segments). In **(B)** the pcer diencephalic branches are visualized after graphically removing the caudal part of the hemisphere than normally hides them (the floating caudal contour of the eliminated part of the hemisphere was drawn in as a curved line extending from the occipital pole to the temporal pole, for reference; a deeper blue distinguishes the cut surface at the telencephalic pallium; the section across the lateral ventricle appears in black); the diencephalon thus liberated is shown undeformed according to the prosomeric model in Figure 1A, so that its PT, Th and PTh regions are seen in their original relationships. The pcer can be seen first contouring the basal peduncle dorsalward in front of the midbrain, and then bending rostralwards along the ventral part of the diencephalic alar plate; it appears cut off at the point where it would enter lateralwards and caudalwards its telencephalic P3 segment (seen in **D**). Two thalamic perforating arteries are represented (tth, thp), jointly with examples of non-perforating va/da thalamic branches of the P2 pcer (thg, pmch). It is not yet known whether there exist also pretectal and prethalamic perforating arteries. In addition, postulated pretectal and prethalamic va/da arteries which may have been misidentified as “thalamic” are also drawn in (see text). The posterolateral chorioidal artery (plch) is a pcer branch that courses dorsally next to the interthalamic zona limitans boundary (passing rostral to the thalamic lateral geniculate primordium; LG) and reaches the chorioidal roofplate of the prethalamus. The latter is continuous caudally with the thalamic one (served by the pmch) and rostrally with the telencephalic counterpart (served by the ach). Compare the thalamus pattern in **(B)** with the schematic cross-section in **(C)**. **(C)** Schematic section transversal to the thalamic neuromere, visualizing its floor, basal, alar and roof plates, jointly with its main arteries. The thalamus lies in the alar domain, capped by the habenula (Th, Hab); the basal domain represents the p2Tg field. Perforant vessels such as tth (from pc) or thp (directly from pcer P1 segment) penetrate the p2Tg (tth rostrally to thp) and then course periventricularly into the alar thalamus, where they serve different polar or paramedian deep populations. The superficial thalamic nuclei are served instead by direct va/da (thg) branches of the rostrally oriented pcer, as well as by collaterals of the pmch artery (pcer) reaching the habenula and the chorioidal plexus of the 3rd ventricle. The LG also receives irrigation from the ach artery, *via* its recurrent thalamic branch (rth, ach, LG; in **B**). **(D)** Schema of the interhemispheric telencephalic face after removing graphically the diencephalon and hypothalamus (interventricular foramen in black; if), showing the main arterial vessels covering this area. The cortex appears color-coded as depending either on the acer (pale yellow territory) or on the pcer (pink territory). The acer gives out orbital (orb), frontopolar (fpol) branches, as well as the terminal pericallosal (pec) and callosomarginal (cmarg) arteries, which produce other frontal and parietal ramifications at the convexity. The pcer gives out its temporo-hippocampal branch (temp) and calcarine (calc) and parieto-occipital (pao) branches. We also see represented the dual irrigation of the chorioidal plexus of the lateral ventricle. This occurs *via* two vessels entering the chorioidal fissure, which stretches from the roof of the interventricular foramen until the uncal pole of the sphenoidal ventricular horn. The ach arises directly from the internal carotid (ic) and enters the uncal tip of the fissure, distributing to the sphenoidal or telencephalic portion of the lateral plexus, which ends roughly under the callosal splenium. In contrast, the plch arises from the pcer, and contours the whole surface of the prethalamus (removed graphically) until reaching the prethalamic supracapsular part of the lateral plexus, which extends from the foramen to the area under the splenium, where it may anastomose with the ach plexus portion. Each of these arterial chorioidal territories has its own venous outflow.

**Figure 9:**
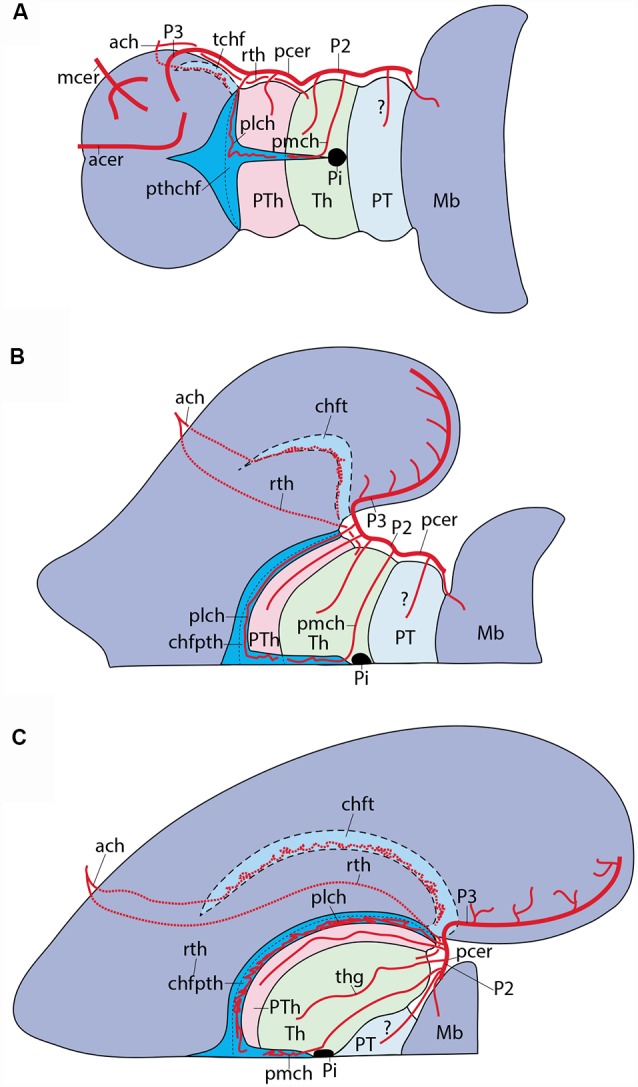
**(A–C)** Schemata of dorsal views of the forebrain, representing three arbitrary developmental stages in which the diencephalo-telencephalic transition evolves dramatically due to the massive growth of the telencephalon, accompanied by disproportionate growth of the thalamus (Th) with respect to pretectum (PT) and prethalamus (PTh) in the diencephalon. **(A)** Initial stage, shortly after telencephalic evagination begins. A color code was applied to the three diencephalic alar territories, and the visible chorioidal roof mainly associated to the prethalamus was represented in brilliant blue, while the final telencephalic portion extends out of our view in all three schemata (seen by transparency in pale blue) into the primitive posterior pole of the hemisphere (the uncus). The pcer artery is disposed longitudinally along the whole alar diencephalon and finally reaches its telencephalic terminal region. We see also the acer and the mcer reaching independently their respective fields. Pretectal, thalamic and prethalamic alar branches arise sequentially from the pcer, as it contours each neuromere, and we see also the pmch reaching the thalamic roof and the plch reaching the prethalamic roof. Separately, the ach reaches the caudal tip of the telencephalic chorioidal fissure (by transparency). **(B)** At this stage, the hemisphere starts to bulge caudalwards and the thalamic mass grows jointly with the internal capsule as it bridges the hemispheric stalk in front of the prethalamus. This development start to cause a stretching of the prethalamus and its associated chorioidal formation (pink plus bright blue). All the vessels are absolutely passive in this process and simply adapt to the emerging new increasingly compressed position of the lateral face of the diencephalon and the stretching consequent to the growth of a larger telencephalic mass. **(C)** At this nearly final stage, the deforming process has brought the lateral diencephalic surface to a transversal topography (90° from its primitive position in **A**). The pink and bright blue prethalamic region is enormously stretched and thinned out, but it still occupies the interface between the telencephalon and the thalamus. The pretectum results partly hidden, but also remains in its original caudal position. The prethalamic chorioidal plexus served by the plch participates in the upper supracapsular part of the fissure (bright blue), having reduced its preforaminal portion and increased in length (by stretching its postforaminal portion); the telencephalic chorioidal plexus served by the ach appears stretched out (pale blue; still by transparency), but essentially in the same position as before. The pmch serves the small thalamic chorioidal plexus in front of the pineal (Pi). As a consequence of such morphogenesis, the pcer seems to have lost its longitudinal P2 course, but topologically this course continues to be present.

**Figure 10:**
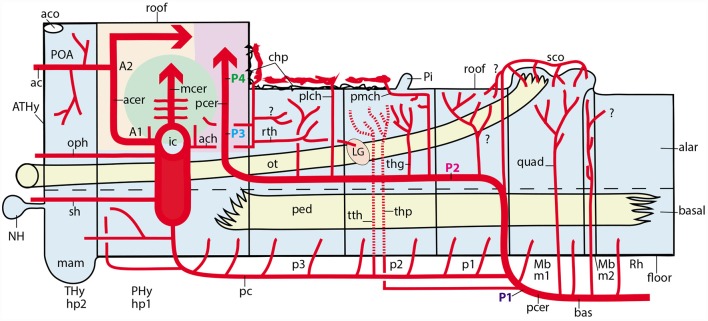
Schematic topologic representation of known or newly postulated forebrain arterial vessels mapped upon the prosomeric model. As regards the topologic forebrain map, which essentially reproduces the semi-realistic version of Figure 1A, its axial dimension has been straightened [elimination of the cephalic flexure, straight floor, straight alar-basal boundary (dash line), nearly straight roof (this has a step as the evaginated telencephalon is reached, for clarity, but even this might be straightened out), and, accordingly, the basal and alar plates also are straight]. Reference structures such as the cerebral peduncle (ped) and the optic tract (ot) are straight or nearly straight. All neuromeres and interneuromeric borders are orthogonally transversal to the axial dimension. In these conditions, it is possible to represent faithfully spatially oriented structures such as the arteries. Dorsal is the direction into the roof, while ventral directs into the brain floor; rostral lies to the left, and caudal to the right. The main subarachnoidal vessels serving this territory derive from the ic, pc, pcer, and bas arteries. One should first examine these fundamental vessels. The ic courses transversally in ventrodorsal direction next to the PHy (crossing the ot); it is thus parallel to the peduncular hypothalamic sector–not shown- tagged as PHy). Its major terminal branches entering transversally into the telencephalon overhead are the acer and mcer vessels, positioned in the map as corresponds after flattening the hemisphere (there is a yellow/green color code for the acer and mcer fields). The posterior telencephalic field is covered by the final, similarly transversal, segment of the pcer (pale violet code). The thick arrows in each case represent simplified pallial arborizations, whereas central branches to the subpallium appear as thin collaterals. The acer also gives out the ac artery which importantly serves the preoptic (POA) and septal regions (the septum lies near the telencephalic roof, paradoxically, and surrounds the anterior commissure, aco, which fate-maps as the rostral end of the roof). The median front of the forebrain is given by the acroterminal preopto-hypothalamic domain (ATHy). Note the optic chiasma (unlabeled) and the neurohypophysis (NH) lie at alar and basal levels of this acroterminal area, respectively. The sh and oph branches of the ic are thus longitudinal arteries. The pc vessel arises from the ic and then topologically descends first along the PHy and then bends caudalwards into a longitudinal para-tegmental course until it meets the pcer near its origin from the bas. Our topologic straightening of the normally bent length dimension has caused the pc to appear as long as it topologically is, though this is not seen in the unstraightened brain, where we mostly see its short transversal hypothalamic course. The pcer continues bilaterally the median bas artery but changes its relative position by contouring dorsalward the peduncle (in front of the midbrain) into a ventral alar level, which it then uses to extend rostralward (longitudinally) until it enters into the telencephalon. This is the basic layout. The midbrain thus appears as a transitional caudal forebrain domain where the vascular patterns gradually change from typical hindbrain features to typical diencephalic characteristics. This again apparently changes when we arrive at the secondary prosencephalon, where our analysis was handicapped by scarce and confusing data (this is the less detailed part of our vascular map, but it can be developed in the future). One fundamental pattern that is pretty clear is that the brain basal plate is irrigated separately from the larger alar plate. A multiplicity of basal (mediobasal or laterobasal) arteries enter the basal tegmentum at all neuromeric levels, as predicted originally by His (1895, 1904) and as expected by the prosomeric model (not so the columnar model, which predicts that basal arteries should extend through the acroterminal dimension into the subpallial telencephalon; there is no sign of that). These basal plate vessels arise sequentially from the as, ve, bas, pcer (P1) and pc arteries. With exception of the thalamic perforant arteries (tth, thp; seen by transparency), which first behave as basal vessels, but then extend intraneurally into the alar domain, a separate set of arteries address the hindbrain, midbrain and diencephalic alar plate. In the hindbrain a pattern of ventroalar and dorsoalar arteries arising from the bas or ve vessels (commonly known as short and long circumferential branches) is clearly repeated, even when some segmental vessels add a jump into the overhanging cerebellum, a morphogenetic deformation (pic, aic, sc). Most dorsoalar hindbrain arteries may give out chorioidal branches. The midbrain also has dedicated alar arteries, such as the quad at the m1 mesomere, possibly duplicated at the m2 companion segment; they arise from the bas or pcer P1. The map next shows that the diencephalic alar plate is covered by successive neuromeric alar branches of the pcer, some of which (in p2 and p3) are chorioidal branches. The pattern thus has changed by moving the bas-like pcer bilaterally to a longitudinal course which is displaced to an alar topology (compare Figure 8C). Apart the midbrain basal branches of the pcer P1, diencephalic basal branches largely originate from the pc artery. The map also places the route and ending sites of the pmch and plch arteries, in contrast with the ach artery, which oddly also produces a recurrent thalamic branch (rth) which targets the lateral geniculate body by extending longitudinally, but backward, into at least the prethalamus and the thalamus.

**Figure 11:**
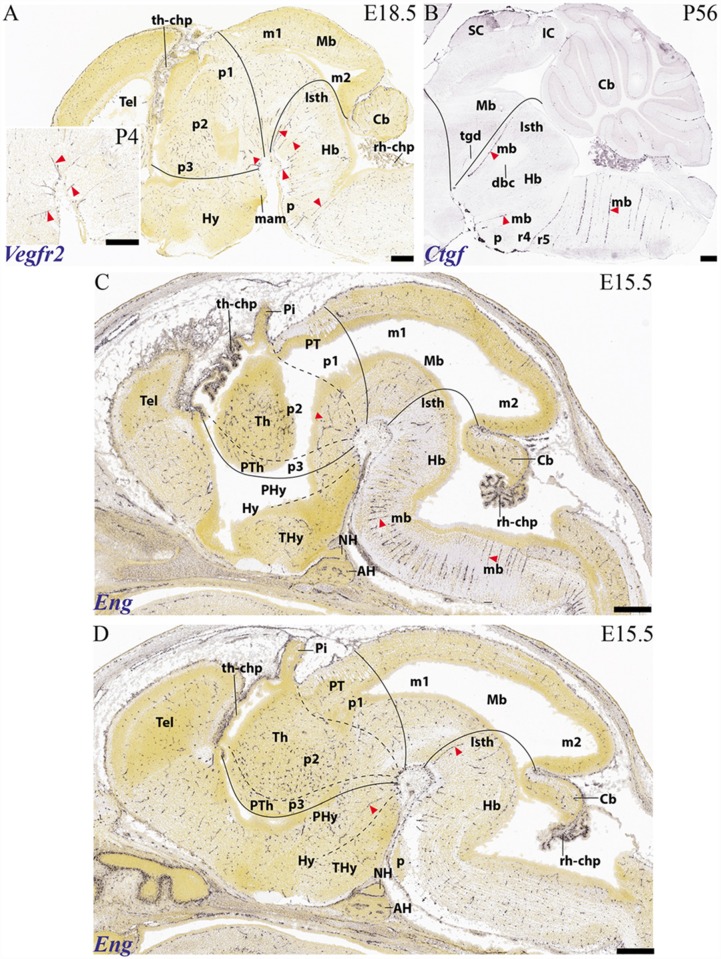
Examples of basal arteries labeled with different gene markers in paramedian sagittal sections (material downloaded from the Allen Developing Mouse Brain Atlas). The illustrated vessels distribute along topologically transversal courses into different neuromeric units of the hindbrain and/or forebrain (major limits indicated by black lines; diencephalic and secondary prosencephalic interneuromeric limits marked by dash lines). **(A)** E18.5 mouse embryo, *Vegfr2* labeled arteries (red arrowheads; inset shows higher magnification detail of cephalic flexure). **(B)** Adult mouse, *Ctfg* label, some radial hindbrain mediobasal arteries (red arrowheads caudal to the isthmo-mesencephalic boundary; black trace; check Mb; Isth). **(C)** E15.5 mouse embryo, *Eng* label, red arrowheads pointing out mediobasal vessels in the thalamic and hindbrain basal plate. Note also chorioidal plexi (th-chp; rh-chp). **(D)** E15.5 mouse embryo, *Eng* label, red arrowheads pointing out mediobasal vessels in the peduncular hypothalamus and the isthmic rhombomere.

Using for simplicity semi-realistic lateral-, medial- and dorsal-view schemata based on a rodent brain (Figures 8, 9), it is feasible to produce a systematic semi-topological classification of the known arterial vessels relative to the prosomerically subdivided surface of the brain. Surface regions represent so many radial histogenetic units reaching in depth the ventricle (presumed mantle layer course of radially penetrating vessels; Figures 1C,D). We left aside for the moment the venous vessels, which are nevertheless susceptible of the same approach (e.g., Padget, 1948, 1957). Some points posed technical difficulties, because some brain portions are grossly deformed morphogenetically in rodents and humans, and may show vascular positions in the adult that do not seem similar to the original embryonic ones. Some extrapolation had to be applied. We also attempted a less realistic, more topological schema (Figure 10), and checked at the Allen Developing Mouse Brain Atlas^1^ the predicted vascular branch trajectories detected by various vascular gene markers (Figure 11). The present results are just a first approximation to this new mapping approach.

Theoretically, the approximation courses through the arachnoid layer are expected to be either longitudinal (i.e., parallel to the brain length axis, which we must remember is sharply bent ventralward at the cephalic flexure; Figure 1A), or transversal (orthogonal to the brain axis and parallel to the changing DV dimension of the neuromeres; Figure 1A). Significant contradiction of our expectations would emerge if oblique vascular courses are found. Some vascular arbors are quite complex, as exemplified by the posterior cerebral artery, which ends in the temporo-occipital telencephalic cortex, but also gives branches to chorioidal roof specializations, as well as to ample alar and basal diencephalic and midbrain areas. The issue will be also touched below whether some vessels on occasion jump from one brain subdivision to another (e.g., from the hindbrain medulla to the cerebellum, implying mixed coverage of different neuromeres).

#### The Vertebral and Median Basilar System

The vertebral arteries (ve) converge into the basilar trunk (bas) approximately at r5 level [producing there also the median descending anterior spinal artery (as)]. The median basilar artery thereafter courses longitudinally along the pontine (r4-r2) and prepontine (r1-r0; r0 = isthmus) hindbrain levels (bas; Figure 8A) up to its final bifurcation into the right and left posterior cerebral arteries (pcer) just beyond the midbrain m1 prosomere (marked by the oculomotor nerve root). Along this median course, numerous *paramedian radial arteries* are produced which penetrate transversally the medial basal plate of the pontine and prepontine rhombomeres all the way to the ventricle (paramedian or mediobasal pontine arteries; mb; Figures 1D, 8A; note much conventional anatomy wrongly ascribes prepontine hindbrain structures to the midbrain; Puelles, 2016; see also Puelles, 2019 [this book] on neuroanatomic terminology). Paramedian or mediobasal penetrating branches of the anterior spinal artery (as) also show the same basal plate related course for the medullary rhombomeres (r5-r11; *mediobasal medullary arteries*; mb; Figures 1C, 8A). At medullary levels, we see also *lateral paramedian or laterobasal* branches of the vertebral arteries, which penetrate lateral parts of the medullary basal plate (e.g., passing through the migrated inferior olives; see lb; Figure 1C). Similar transversal neuromeric medial branches arise from the rostral end of the basilar artery (bas), the origin of the posterior cerebral artery (pcer), or the posterior communicating artery (pc), and penetrate in essentially the same radial way the interpeduncular surface of the prepontine hindbrain (e.g., level of interpeduncular nucleus), the midbrain and the posterior perforated space rostral to the oculomotor nerve roots (diencephalic in nature). There are specific isthmic basal branches of the basilar artery, which we found labeled with the *Ctfg* and *Eng* markers in the mouse (mb; Isth; Figures 11B,D). The more rostral medial branches reach directly the basal plate domains of the diencephalic neuromeres and even the retromamillary hypothalamic area, which corresponds to the peduncular basal hypothalamus (hp1; Figure 1A; compare Figure 8A, and vessel marked with red asterisk in Figure 11D). It is not clear so far whether similar medial branches penetrate the mamillary region of the terminal hypothalamus, which is postulated in the prosomeric model as the rostralmost basal plate territory (mam; Figure 1A). This area borders the acroterminal hypothalamic region, which represents the rostral median end of the forebrain, and extends between the mamillary body and the anterior commissure, including unique basal formations such as the tubero-mamillary area, median eminence, infundibulum and neurohypophysis, and unique alar formations such as the optic chiasma, the preoptic lamina terminalis and the anterior commissure (Puelles et al., 2012a; Ferran et al., 2015c; Puelles and Rubenstein, 2015). This somewhat “special” rostromedian territory seems to receive direct branches from the internal carotid (e.g., the superior hypophysial artery, and the ophthalmic artery), or branches from the anterior communicating artery (sh; oph; ac; Figures 8A,B). In this domain the vessels usually penetrate along radial lines approaching the ventricle in curves best observed in horizontal sections (e.g., Puelles et al., 2012a, their Figure 8.12).

Apart of these clearly transversal and segmental medial paramedian or mediobasal arteries, lateral branches of the basilar and vertebral arteries follow analogous but longer parallel courses relative to the DV dimension of all rhombomeres in order to serve their alar plate territories through alar entrance points (e.g., pic; Figure 1C; bas branches in Figure 1D). To this end, they contour superficially the hindbrain basal plate domain and then penetrate either ventrally or more dorsally the alar plate domain. One of these lateral alar arteries is the *postero-inferior cerebellar artery*, which we judge to parallel r9 in its transversal dorsalward approach to the medullary sensory centers and its subsequent jump into the caudal cerebellum, giving other branches to the posterior spinal artery, and the IV ventricle chorioidal plexus (pic; Figures 1C, 8B). There are also several so-called *lateral medullary arteries* related to r6-r8, which we classify as ventral and dorsal alar vessels (va, da; Figure 8B). The human *antero-inferior cerebellar artery*, seems to run dorsalward transversally along the r4/r5 boundary, or next to it (aic; Figure 8B); indeed, it reportedly passes rostral to the abducens nerve root in r5 and caudal to the facial and stato-acoustic nerve roots in r4, giving alar plate branches complementary to those of the pic. A similar antero-inferior cerebellar artery with identical neuromeric topography exists in the mouse, which serves a large part of the IVth ventricle chorioidal plexus and then jumps into the caudal cerebellum (r1; Scremin and Holschneider, 2012). The *lateral short and long circumferential pontine arteries* are also ventral and dorsal alar branches of the basilar artery at pontine levels, corresponding at least to r3 and r4 (va, da; Figures 1D, 8B), but possibly also to r2 and r1 (since the pontine formation partly covers these domains as well; see Watson et al., 2019, this book). We did not find useful human data specifically on r2 and r1 vascularization (apparently, these domains were not recognized as distinct regions in conventional columnar neuroanatomy), but we expect that these neuromeric units (important because they hold most of the principal sensory and motor trigeminal nuclei, apart of vestibulocochlear centers; Puelles, 2013) are also served in their alar domains by segment-specific *lateral (ventral and dorsal alar) circumferential prepontine arteries* that probably have been observed, but were misclassified as “pontine” (va, da; Figure 8B). The isthmus or r0 level is characterized by the well-known *superior cerebellar artery*, an alar plate targetted vessel which approaches the cerebellum through its rostral end, topologically associated to the isthmus-derived vermis, but possibly also crossing into the r1-related paramedian hemisphere (sc; Figure 8B; this description agrees with the medial and lateral branches of the mouse superior cerebellar artery; Scremin and Holschneider, 2012).

#### The Posterior Cerebral Artery System

The pcer artery diverges sharply from the bas course, since it follows a topographically transversal course lounging ventrodorsally the pes pedunculi in front of the midbrain, reaching the local alar plate (it thus behaves as a circumferential vessel with regard to the peduncle). The initial pcer is conventionally divided into segments P1 and P2 by the confluence or origin of the posterior communicating artery; the latter is often interpreted developmentally as a longitudinal descending branch of the internal carotid (Padget, 1948). The pcer extends beyond the midbrain and ends serving temporal and occipital cortical territories (segments P3 and P4). The P1 segment of the pcer gives rise to some medial (interpeduncular) *midbrain* and *diencephalic* *basal* *branches* (mb-P1; Figure 8A), plus the *quadrigeminal* and *thalamo-perforant arteries* which target parts of the alar plate (in human; see Scremin and Holschneider, 2012). The large alar collicular plate of the midbrain is partly served by the quadrigeminal artery, also a “lateral or ventral alar artery” targeting alar domains (Figure 8B), and partly by a dorsal alar, supracollicular network of unclear origin (Scremin, 2015). In contrast to most other alar plate-irrigating vessels treated here, the thalamo-perforant or inferior thalamic artery apparently enters the brain through the posterior perforated space (presumably through the basal plate of the thalamic p2 prosomere; thp; Figure 8B), and then follows a deep ventrodorsal penetrant course next to the ventricular lining until it reaches the medial thalamic region in the alar plate (this deep course is similar to that of other perforating thalamic branches issued by the posterior communicating artery; see pc; Figure 8C; Salamon, 1971, 1973; Lazorthes et al., 1976; Percheron, 1976a, b; Haines, 1997; Duvernoy, 1999; Naidich et al., 2009; Ten Donkelaar, 2011).

As mentioned above, the posterior communicating artery may be understood either as a descending branch of the internal carotid or as a bilateral rostral basal extension of the pcer. It contributes distinct mediobasal branches for the diencephalic and hypothalamic prosomeres and provides also perforating thalamic arteries at p2 level (see “Discussion” section). Otherwise, the pc does not seem to produce alar plate branches (Figure 10).

The P2 segment of the pcer reportedly produces various posterolateral alar thalamic branches (the *thalamo-geniculate arteries*) which invade the anterolateral part of the thalamus and contribute to parts of the pulvinar and the geniculate bodies (thg; Figures 8B,C, 10; Lazorthes et al., 1976; Percheron, 1976a, b; Ten Donkelaar, 2011). Other less evident pcer branches possibly penetrate similarly the pretectum and caudolateral parts of the prethalamus (Salamon, 1971, 1973). Neither the general nor the specialized literature mentions the pretectum nor the prethalamus as regards vascularization, but we know they are differentially vascularized, as we saw in the first part of this report. According to Puelles and Rubenstein (2003), we call “prethalamus” the classic “ventral thalamus.”

As a consequence of differentially massive thalamic and telencephalic growth, the prethalamus, lying intercalated between telencephalon and thalamus (PTh, Th; Figure 1A), becomes flattened between them. The classics referred systematically to a so-called “thalamo-striatal” interface (also to the thalamo-striate sulcus, a.k.a. sulcus terminalis) as representing the tel-diencephalic border. According to present embryological knowledge, this boundary is neither thalamic nor striatal, since the *prethalamus* prosomere takes the thalamus’s position on the diencephalic side, and the *medial ganglionic eminence*—MGE- takes the striatum’s—LGE- position on the telencephalic side). Moreover, the MGE has been subdivided recently into parallel pallidal and diagonal area components (Puelles et al., 2013, 2016; see also Flames et al., 2007). The latter component, the *diagonal area*, seems to be the subpallial element that contacts with the prethalamus across the sulcus terminalis (the diagonal area is a full radial histogenetic domain that comprises the diagonal band nuclei at the surface, the substantia innominata, basal nucleus of Meynert and the internal pallidum at intermediate mantle levels, and the medial part of the supracapsular bed nuclei of the stria terminalis at periventricular level). The main prethalamic cell masses are the superficial pregeniculate and subgeniculate visual nuclei (lying next to the thalamic lateral geniculate nucleus), the reticular nucleus and the zona incerta complex, plus the prethalamic eminence (classically misnamed “thalamic eminence”). Increasing the confusion, the literature sometimes wrongly ascribes some of these entities to an outdated category, the subthalamus (review in Puelles et al., 2012a).

The existence of at least one alar prethalamic artery branching out from the pcer at the end of its P2 segment is thus a distinct possibility (Figure 10), though it might originate alternatively from the posterior communicating artery, or from the tubero-mamillary artery (see “Discussion” section on this vessel) that serves the anterior thalamic pole; the *posterolateral chorioidal artery*, branch of the pcer, possibly represents a good candidate for the missing alar prethalamic branch (see below). A further possibility is that the alar prethalamus is partly served by the anterior chorioidal artery, since this is described to reach with its branches the lateral geniculate body, implying that it would have to cross first the prethalamus (ach; plch; LG; Figures 8B, 10; see “Discussion” section).

The pcer also emits within its P2 (diencephalic) segment branches for the diencephalic dorsal alar and roof neighborhoods, including the *posteromedial chorioidal artery* serving the pulvinar, geniculate bodies, habenula and the thalamic chorioidal plexus of the 3rd ventricle, and the mentioned *posterolateral chorioidal artery* for the prethalamic eminence and the prethalamic participation in the supracapsular part of the chorioidal fissure and corresponding lateral ventricle chorioidal plexus. The plch was reported by Stewart (1955) to ascend transversally along the zona limitans intrathalamica (the prethalamo-thalamic boundary; see plch; Figure 8B). It serves selectively the part of the lateral ventricle chorioidal plexus that extends caudolaterally from the interventricular foramen, along the thalamic chorioidal sulcus, and ends at the begin of the sphenoidal ventricular horn, roughly alevel with the lateral adult geniculate body (see plch; pmch; ach; Figures 8C, 9A–D).

The topology of all the P2 pcer branches is difficult to visualize in human material even after careful dissections, due to their apparently indiscriminate collection within the deep and narrow arachnoid pocket that separates the medial aspect of the temporal lobe from the backwards-oriented (originally lateral) diencephalic surface and the midbrain (see progressive diencephalic deformation affecting thalamus and prethalamus, as well as the corresponding chorioidal telae, in Figures 9A,B,D). We deduce that after coursing topologically *rostralwards* along the whole primitive lateral aspect of the diencephalon, producing relevant segmental alar branches for the three diencephalic segments (Figures 9A,B,D), the pcer starts its P3 segment as the artery reaches the temporal lobe of the telencephalon close to its uncal pole (Haines, 1991, 1997). There it produces its anterior and posterior temporal branches, including secondary uncus, amygdala and hippocampal branches. The final P4 segment gives rise to parieto-occipital and calcarine terminal branches (Figure 8D).

#### The Internal Carotid System and the Middle Cerebral Artery

We estimated the topological position of the internal carotid artery syphon relative to the brain surface as ascending from basal into alar regions along the peduncular hypothalamus (the artery passes early on in development behind the eye vesicle and stalk, both derivatives from the terminal hypothalamus, and its major terminal branches serve the evaginated telencephalon). Accordingly, it crosses orthogonally the longitudinal optic tract (ic; ot; PHy; THy; Figure 10). In contrast to the visualization problems posed by the pcer, the internal carotid and its main collateral and terminal branches seem rather straightforward, since both the telencephalic subpallium (except POA) and pallium are dorsal derivatives of the same prosomere (hp1, ic; Figure 10). However, as we will see, the ic also gives collateral branches into the THy, as well as recurrent collateral branches into the diencephalon. We think that the *superior hypophysial artery* (sh) is given out as a rostrally directed longitudinal branch while the ic is passing next to the hypothalamic basal plate region; this branch would have to grow strictly lengthwise from PHy into the THy region to reach the neurohypophysis (sh; Figures 8A,B, 10). This agrees with the basal position of the neurohypophysis (Puelles et al., 2012a; Puelles and Rubenstein, 2015). On the other hand, the *ophthalmic artery* (oph) is also a rostrally directed longitudinal branch that arises instead from the ic at alar plate level of PHy, and it invades longitudinally the eye, a THy derivative (oph; Figures 8A,B, 10). The ic also gives out two caudally directed or recurrent longitudinal branches.

One of them is the *posterior communicating artery* (pc), which clearly arises from the ic at alar levels of the forebrain (topologically dorsal to the optic tract; embryonic analysis—e.g., Padget, 1948—confirms that it is a descending or recurrent branch of the ic, not a branch of the pcer). The pc first courses ventralwards (crossing the optic tract) along the dorsoventral dimension of the peduncular hypothalamus. Here several basal or tegmental branches are given for both PHy and THy. After it reaches the cephalic flexure, it arches longitudinally backwards under basal p3 and p2 until it meets the pcer at roughly p1 level (see Stewart, 1955). Along this longitudinal segment the pc gives out sequentially 7–8 tegmental branches for the hypothalamus and the three diencephalic neuromeres, a pattern that corroborates its local longitudinal nature (pc; Figure 10; these branches are confusingly known as *posterolateral central arteries*, which complement the analogous *posteromedial central arteries* originated from the pcer; Ten Donkelaar, 2011; his Figure 2.10). It would be clearer terminologically to call them *hypothalamic basal* and *diencephalic basal* arteries, since the prefix “postero-” used for both pc- and pcer- basal branches is ambiguous as to their specific origins, and the “central” descriptor appears in a different usage for deep branches of the cerebral arteries entering the telencephalic subpallium, a property not shared by these selective forebrain basal plate vessels. In the human brain the implicitly bipartite bent course of the pc (first transversally dorsoventral across the optic tract and then longitudinal under the diencephalic tegmentum) results secondarily straightened out due to massive growth of the peduncle, so that in the usual basal images of the polygon of Willis the pc seems to course in a straight line orthogonally to the optic tract.

The literature mentions among the “pc central branches” a distinct “tubero-thalamic” perforating artery (tth; Lazorthes et al., 1962, 1976; Plets et al., 1970; Percheron, 1976a), which is contradictorily represented by Ten Donkelaar (2011; his Figure 2.20) as a branch of the pc arising midways along its longitudinal trajectory. We think, consistently with this drawing, that it probably penetrates ventrally the p2 tegmentum and takes internally a deep perforating dorsalward course into the anteromedian pole of the (alar) thalamus (tth, pc; Figures 8B,C, 10; it serves there the anterior thalamic nucleus and the polar part of the ventral anterior thalamic nucleus). However, the description of this artery in the Ten Donkelaar’s (2011) text is confusing, since it is defined as a “*premamillary* (anterior thalamoperforating or tubero-thalamic) artery,” whose territory includes the *posterior part of the optic chiasm, the optic tract, the posterior part of the hypothalamus with the mamillary body*, *and the reticular nucleus* *of the thalamus*, apart the terminal thalamic arborization (data attributed to Plets et al., 1970; Percheron, 1976a). A premamillary-thalamic or tuberal-thalamic course might allow the collateral vascularization of the chiasma and mamillary region, but does not agree at all with the perforating tegmental course *depicted* by Ten Donkelaar (2011; his Figure 2.20a,b), which clearly implies a penetration caudal to the retromamillary area (remember the ic correlates topologically with the retromamillary PHy; Figure 10). Our analysis suggests that this perforating artery probably relates primarily to the p2 (thalamic) diencephalic neuromere, which contains the thalamus in its alar domain. Once the pc branch has entered the thalamic tegmentum, it might give out rostrally coursing hypothalamic branches that reach the mamillary body and even the more distant optic chiasma *after crossing the prethalamic, retrotuberal and tuberal tegmentum*. Additional dorsally perforating branches for the *alar prethalamic* reticular nucleus might arise as well. However, we do not have any positive evidence corroborating such hypothalamic or prethalamic branches of the tth, as illustrated in the literature. The “tubero-” root in the name of this thalamic artery apparently refers explicitly to a penetration through the *tuberal region*, or perhaps the *tubero-mamillary* *area* (the tubero-mamillary area lies between NH and mam in Figure 10), but it does not seem plausible that a branch of the pc enters so far rostrally into the forebrain basal plate to finally reach the thalamus. A possibly satisfactory resolution of this semantic conundrum is that the “tubero-” root in the name possibly refers instead to the “posterior tuberculum,” an old anatomic term used for what we now conceive as the thalamic (p2) tegmental region (e.g., see such use in Puelles et al., 1987). The course of the tth depicted by Ten Donkelaar (2011) would agree with the alternative name we propose—“*tuberculo-thalamic perforant artery*”—which would describe perfectly this vessel. We suggest that, unless strong evidence for a straightforward tuberal entrance of this vessel into basal terminal (premamillary) hypothalamus is available, or is newly found, its name should be changed to “*tuberculo-thalamic perforant artery*.” Whether it is true (and not a myth based on a semantic error confusing “tubercular” with “tuberal”) that parts of the tuberal and chiasmatic hypothalamus and of the prethalamus are served by the tth will need renewed research. We would not be surprised if the alar prethalamus is found to receive an analogous “tuberculo-prethalamic perforant artery,” branching off directly from the pc or from the root of the tth.

The ic next produces another recurrent collateral branch, the *anterior chorioidal artery* (ach), normally originated shortly above the pc. The ach arises within the telencephalic region, since immediately it gives out “central” collaterals to pallidal and diagonal (i.e., innominate area) subpallial regions, and then follows in the subarachnoidal space the hemispheric sulcus (the classic tel-diencephalic border) until it reaches the roof-plate-related tip of the chorioidal fissure, where it dips into the telencephalic part of the lateral ventricle chorioid plexus (ach; chp; Figures 8C, 9, 10). Before the embryonic ach reaches its chorioid target, it produces a longitudinally descending (recurrent) diencephalic collateral that eventually reaches beyond the prethalamus (alar p3) the neighborhood of the lateral geniculate primordium in the thalamic (alar p2) lateral wall (ach; LG; p3, p2; Figure 10; Stewart, 1955). This implies that this recurrent branch of the ach enters the alar diencephalon following longitudinally the optic tract across the lateral surface of the prethalamus, in order to reach the lateral geniculate nucleus (LG) present at the primitive lateral surface of the thalamus proper (ach; p3; p2; LG; Figure 10; we showed in Figure 9 the subsequent deformation of this lateral diencephalic wall carrying the LG). Various authors have affirmed that the ach participates at least partially in the vascularization of the LG and other neighboring superficial nuclei (e.g., Stewart, 1955; Salamon, 1971, 1973; Tatu et al., 1998, 2001; Tatu’s relevant mappings are reproduced as Figures 2.7b, 2.8a,b, and 2.9a in Ten Donkelaar, 2011). Unfortunately, these sources do not mention whether the same recurrent branch of the ach also vascularizes *en passant* the neighboring prethalamic (ventral thalamic) retinorecipient centers (pregeniculate and subgeniculate nuclei), and/or more deeply the reticular nucleus, which lies next to the substantia innominata, as would be possible (see “Discussion” section). Additionally, Salamon (1971) states that the ach artery (actually its recurrent diencephalic branch) also serves part of the pes pedunculi, including the substantia nigra. Importantly for our topologic mapping, these two entities reside in the basal plate (Figure 10). Since the peduncle extends all the way from the hypothalamus into the pons (ped; Figure 10), and the substantia nigra is mes-diencephalic (Puelles et al., 2012b; Puelles, 2016), we interpret that Salamon (1971) probably referred to prethalamic and thalamic parts of the diencephalic tegmentum and associated parts of the substantia nigra (rather than more caudal pretectal and midbrain parts). These basal loci may be served by transverse collateral tegmental branches arising from the recurrent ach diencephalic branch as it courses caudalwards along the alar p3 and p2 territories. However, the objective evidence for these details is poor so far.

As represented in Figure 10, the *anterior chorioidal artery* is thus basically a telencephalic roofplate-targeting, dorsalward growing branch of the ic that courses dorsalward (transversally) along the interneuromeric hemispheric sulcus (hp1/p3 boundary). It finally participates in the temporal (telencephalic) part of the lateral ventricle chorioidal plexus. Somewhat surprisingly, it also turns out to give out a longitudinal recurrent diencephalic branch apparently serving superficial retinorecipient parts of prethalamus and thalamus (this branch has been illustrated by Padget, 1948 and Stewart, 1955). This strictly collateral vessel which advances in a wholly different direction (Figure 10) is routinely referred to also as the “anterior chorioidal artery,” though its specific thalamic (and tegmental) target is not chorioidal at all, producing confusion in the reader (see “Discussion” section). It would be convenient to give this longitudinal vessel a distinctive name—perhaps “*recurrent thalamic artery*”—understanding it as a branch of the ach.

In the human brain the ach proper enters the uncal end of the chorioidal fissure found at the temporal (sphenoidal) tip of the lateral ventricle, and it serves the sphenoidal part of the lateral chorioidal plexus along its parafimbrial course (irrigating also adjoining subpallial elements such as the tail of the caudate nucleus and the amygdalar parts of the bed nucleus stria terminalis formation) until the telencephalic plexus meets close to the pulvinar the prethalamic part of the lateral chorioid plexus, served by the posterolateral chorioid artery (from the pcer system). These rather difficult chorioidal relationships are repeatedly schematized in our Figures 8C, 9, 10 as we presently understand them. The literature normally gives a simpler but less satisfying view of these details, because it disregards wholly the existence of the prethalamus, a diencephalic neuromere which unavoidably separates the telencephalon from the thalamus. The prethalamus has its own chorioidal roof plate domain, ampler than that of the thalamus/epithalamus, and this *must* enter into the picture (Figures 8C, 9, 10). As is variously illustrated in these schemata, we hold that the post-foraminal portion of the classical chorioidal fissure which seems attached to the thalamic chorioidal sulcus (next to the mythic lamina affixa) represents actually most of the prethalamic roof plate. Only the terminal portion of the fissural chorioid plexus found along the sphenoidal horn of the lateral ventricle is properly telencephalic (see also, Puelles, 2019; this book). This concept of a double prethalamo-telencephalic nature of the fissural roof plate tela is consistent with the existence of the repeatedly described separate plch and ach arterial peduncles of the two moieties of the chorioidal plexus of the lateral ventricle. Interestingly, Padget (1957) observed likewise two separate chorioidal veins collecting the respective efflux of these two chorioidal capillary plexus domains.

The internal carotid resolves in its two terminal branches, the anterior and middle cerebral arteries (mcer, acer; Figures 8B, 10). These only need to ascend ventrodorsally in a strictly transversal topologic course within the evaginated telencephalic part of the hp1 neuromere (acer; mcer; hp1; Figures 1, 10) into their respective central and superficial target areas. We applied a color code in Figure 10 to delimit schematically the main flattened cortical arterial regions. We think it merits commenting that the mcer seems at first glance to cover only central parts of the hemisphere, but this region actually corresponds to the whole topologic anteroposterior extent, since once the human hemisphere adopts its characteristic inverted C-shape, the mcer field ranges from the frontal lobe at the front, passing through the insula and parietal lobe, to the retrocommissural upper temporal gyri, reaching also the temporal pole, the topological caudal end of the hemisphere (compare Ten Donkelaar, 2011; his Figure 2.5). The cortical areas at the convexity and those occupying the interhemispheric cortex, that is, the cortex lying closer to the septocommissural *roof plate*, are ascribed to the acer and the pcer, with the parieto-occipital fissure as approximate mutual boundary in the human brain. In their initial ascending course, both acer and mcer produce first deep “central branches” for the subpallium, where the recurrent artery of Heubner (acer) and numerous lenticulo-striate arteries (mcer) penetrate the anterior perforated space to serve *via* straightforward radial courses the striato-pallidal basal ganglia within. The schema suggests that the tail of the caudate and the temporal-lobe-related part of the bed nucleus striae terminalis, jointly with the centromedial (subpallial) amygdala, are served by the ach, as commented above, though a contribution from amygdalar branches of the pcer is not impossible.

The acer gives out the *anterior communicating artery* (ac; a rostrally oriented longitudinal vessel extending from a hp1 neighborhood into a hp2-related preoptic zone; ac; POA; Figure 10), as well as its orbital and frontopolar branches, and then immediately proceeds into a cingulate course (pericallosal and callosomarginal branches providing irrigation to the interhemispheric limbic, frontal and parietal lobes and the correlative convexity areas; acer; fpol; pcall; cmarg; Figure 8C). This main course lies parallel to the septocommissural plate (particularly the pericallosal branch), and this implies topologically a final *longitudinal* anteroposterior course inside the cortex, and next to the commissural roof plate, approaching final potential retrosplenial anastomoses with the parieto-occipital pcer branches (acer; pcer; Figures 8B,C, 10).

## Discussion

The two parts of this report will be discussed in the following sections. The comments center on the spatiotemporal and topologic patterns observed, rather than on potential mechanisms, since we have not studied these. We’ll limit speculative comments to a minimum.

### Early Vascular Penetration Patterns in Mouse Embryos

#### Heterochronic Formation of the PNVP

Our data about the timing of PNVP formation in the mouse are roughly consistent with earlier literature cited in the Introduction, as well as with the human studies of Padget (1948) and Stewart (1955). A sizeable PNVP network was present as early as E8.5, and related selectively to alar plate territories of the whole forebrain (albeit only with partial covering of the telencephalic and eye vesicles). In the hindbrain, the PNVP covered the alar plate and a lateral part of the basal plate. The floor plate and the roof plate were distinctly devoid of this formation at this stage. The PNVP ventral boundary related to the molecularly-defined alar-basal border in the forebrain was quite distinct, and could be followed also into the paraneural mesoderm. The vascularly nude ventral neural and mesoderm domains relate topographically to the perichordal environment, which is reportedly rich in chorda-produced and diffused SHH morphogen. The notochord ends rostrally under the mamillary pouch (Puelles et al., 2012a; Puelles and Rubenstein, 2015). Midline SHH concentrations are sufficient to induce homeotically the *Shh* gene at the *floor and basal plate* of all forebrain regions (midbrain, diencephalon, hypothalamus; Martínez et al., 2012; Puelles, 2013), but only at the *floor plate* of the hindbrain. The similarity in the differential forebrain vs. hindbrain spatial SHH and vascularization pattern tempts us to conjecture that the observed early lack of ventral PNVP may be directly or indirectly related to local SHH effects inhibiting vascular sprouting in a given spatial range around the notochord.

It is known as well that the basal plate is the region that most precociously initiates neurogenesis, curtailing early its proliferative growth, whereas the alar plate is retarded in neurogenesis and shows protracted proliferative expansion (Puelles et al., 1987; review in Puelles, 2018). The predominant initial alar distribution of the PNVP thus suggests a relationship with actively proliferating zones of the brain wall with scarce differentiation phenomena. Whether caused by a local notochordal blocking effect, or by a slowed proliferation (or both), the basal retardation is gradually resolved during the following days of gestation, apparently by reduction and physical separation of the notochord from the floor plate, and by parallel circumferential expansion of the primordial alar PNVP into the pial surface of the basal plate (and, ultimately, of the floor plate). In contrast, the paramedian mesoderm continues scarcely vascularized; at E10 and E11.5 the local cellularity has sharply decreased (cell death?), and ample spaces appear fluid-filled; this suggests incipient formation of the basal subarachnoid cisterns (see the cephalic flexure in Figure 6D, and similar images in Figure 7).

#### Heterochronic Formation of Penetrating Vessels (PVs)

The earliest forebrain PVs were already found at E8.5, that is, roughly as reported in the literature, but distantly from the lower medulla, which appears devoid of PVs at this stage. These E8.5 forebrain PVs were found at both sides of the dorsal di-mesencephalic border (DMB), identified by selective alar diencephalic *Pax6* expression (Figures 2A,B). Independently of the initial parallelism of PVs in the pretectum and in the neighboring rostralmost tectal plate, subsequent stages examined showed that PVs in this area rapidly proceeded to cover homogeneously the whole alar pretectum (in an apparent dorso-ventral gradient), but progressed less quickly at the dorsal midbrain. The dorsal tectal plate always showed a marked rostrocaudal decreasing gradient in the number of PVs. We think that this difference in pattern between alar pretectum and alar midbrain is due to the marked proliferative gradient known to occur rostrocaudally across the midbrain, due to reported mitogenic signaling from the isthmic organizer and caudal midbrain *Wnt1* expression (Puelles, 2013).

Why forebrain PVs should first form at the dorsal-most pretectal area and adjoining midbrain is difficult to explain without speculation. There is some amount of proliferative and neurogenetic precociousness associated to this site (the pretectum is the most precocious part of the diencephalon, i.e., is the first neuromere visualized in this area), but neurogenesis seems to start 1–2 days later, which seems to exclude this differentiative process as a causal determinant of the local PVs. This neural tube locus is also particular in developing at the local roof plate both a major commissure (the posterior commissure, which emerges quite early) and a secretory organ (the subcommissural organ), which secretes material into the ventricular fluid that forms the mysterious fiber of Reissner. It may be conjectured that the barely known molecular idiosyncrasy of this environment somehow triggers early sprouting of PVs.

In contrast with the precociously invaded alar pretectum, the alar thalamus remains nude of PVs until E11.5 (3 days later!), while the alar prethalamus displays earliest PVs at E9.5 and soon is profusely penetrated (similar to pretectum, but slightly later). There is accordingly a rostrocaudal gradient in the midbrain and no gradient at all in the diencephalon, whose three neuromeric units display independent heterochronic timetables, all of them apparently unrelated to neurogenetic patterns, since neurogenesis is delayed throughout the whole alar diencephalon (Puelles et al., 1987; Puelles, 2018).

Agreeing with earlier observations, hypothalamic PVs were first observed in the alar hypothalamus at E9.5, rostrally to those in the prethalamus, while the PNVP has not yet covered the hypothalamic basal plate. This alar site lies ventral to the optic stalk area. This points to the prospective subparaventricular area, where the anterior hypothalamic and suprachiasmatic nuclei develop (Puelles et al., 2012a). Separated by the non-vascularized optic stalk and eye vesicle (which only start to be invaded by PVs at E11.5), other more dorsal E9.5 PVs appeared at the preoptic region (telencephalic subpallium). POA is marked selectively by its ventricular expression of *Shh* (Bardet et al., 2010; Puelles et al., 2016), from where tangentially migrating neurons ulteriorly extend into adjacent diagonal and pallidal areas, but not into prospective striatum. Earlier work (e.g., Vasudevan et al., 2008) also identified earlier vascularization of the subpallium compared to the pallium but did not notice that the striatal anlage is relatively retarded within the subpallium. The pallium starts to be invaded by PVs at E11.5, 2 days later.

Our observations indicated a rapid appearance of PVs in the forebrain basal plate between E9.5 and E10. The m1 mesomere shows both basal and alar PVs at E10, as well as an incipient basal PVVP. In contrast, the thalamus still remains vascularly nude at E10. Incipient PVs were also observed at the dorsal part of the hypothalamic basal plate at E10. Dorsally to this locus, the alar hypothalamic areas, and the preoptic, diagonal and pallidal subpallial subdomains were already abundantly served by PVs at this stage, while the more dorsal pallial and striatal regions remained free of PVs in the telencephalon. A subpallial telencephalic PNVP network starts to be developed at about the same stage.

Insofar as there is already precocious *neurogenesis* in the forebrain basal plate at E10, it may be considered that the maturational change in the basal mantle layer may be causally related to the rather sudden retarded appearance of PVs throughout this forebrain longitudinal zone. This pattern also shows spatial correlation with the basal forebrain expression of *Shh* (Figures 6D,F), and no doubt with various other molecular markers typical of this longitudinal zone. Since we previously correlated high perichordal SHH levels (in the mesoderm) with *retardation* of initial basal PNVP formation, but now it seems that the SHH-rich area of the basal forebrain *abruptly develops* PVs, differential effects would be implied. An early *blocking* SHH effect on mesodermal *PNVP formation* may be functionally distinguishable from a *permissive* SHH effect 1.5 days later on intraneural *PV sprouting*. The tubero-mamillary basal hypothalamic area that secondarily downregulates by E10 its early *Shh* expression remarkably remains devoid of PVs (Tu; mam; Hy; Figures 6F,G).

The results obtained at E11.5 reveal a much more homogeneously advanced state of forebrain vascularization as regards the presence of both PVs and PVVP networks practically everywhere, with the exception of the most immature parts of the cortex. The thalamus nevertheless still maintains a relatively peculiar aspect, in displaying mainly PVVP formation, with scarce PVs. This suggests that perhaps PVs penetration is barely starting at this stage, irrespective that a PVVP started earlier elsewhere may have started to expand tangentially into the thalamus (Th; Figure 7B). Such an extrinsic PVVP source of thalamic irrigation probably implies the underlying basal plate, given the correlative unique existence of a *tuberculo-thalamic perforant artery* (a pc branch; formerly probably misnamed as “tubero-thalamic”; see “Results” section) and an *inferior paramedian perforant thalamic artery* (a pcer branch). These singular vessels both penetrate vertically the thalamic basal plate (at the interpeduncular fossa) and then ascend through the periventricular stratum to irrigate alar plate periventricular thalamic derivatives (see Figure 8C). This basal + alar pattern is not seen anywhere else in the brain, with the possible exception of the prethalamic reticular nucleus (see above). A major periventricular part of the thalamus would thus be served directly *across the PVVP* *via* perforant arteries, while the topologically superficial rest of the thalamus would be covered by the thalamo-geniculate arteries and branches of the posteromedial chorioidal artery (pcer system) or the recurrent thalamic branch of the anterior chorioidal artery (ic system). The latter directly penetrate through superficial points the alar thalamus proper.

Observations on the telencephalon at E11.5 show a very significant change. The whole subpallium is now in the midst of massive neurogenesis, as shown particularly by the *Dlx5*-labeled lateral and medial ganglionic eminences (Figure 7F). PVs are now present also in the striatal subdomain (LGE), but in less number than at the Pall-Dg-POA subdomains (MGE), where the PVVP is also better developed (LGE; MGE; Figure 7G). A few PVs are also starting to invade the neighboring pallium, apparently independently from those entering the striatum. The pallial PVs appear dispersed spatially in a ventrodorsal gradient, probably influenced by the larger surface expansion rate of the more immature upper parts of the pallium. *Pax6*-reacted adjacent sections reveal a sharp gradient in the retarded development of the pallial mantle layer (pallium vs. LGE; Figure 7H). Blood vessels have not yet begun to invaginate the thin tela closing the local prethalamic chorioidal fissure to form an incipient chorioidal plexus (chf; Figure 7G).

Analysis of these forebrain results suggests that there is perhaps no simple explanation of the overall vascularization pattern. Alar plate vs. basal plate differences are clearcut and are shared with minor variations by all forebrain segments. This indicates that each one of these longitudinal zones obeys to particular rules as regards both PNVP and PVs formation. The most precocious *alar* PVs do not seem related topographically to sites characterized later by early neurogenesis. We already conjectured that basal PNVP and PV formation may be transiently blocked at early stages, due to direct or indirect chordal effects, or to local absence of VEGF-A. It may be further speculated that the primary cause of a heterochronic pattern of alar vascularization might reside in the emergence of unique regional (neuromeric) alar molecular profiles which modulate differentially not only the timing of intrinsic histogenetic progress within the alar neural wall (e.g., proliferation, neurogenesis and axonal navigation), but possibly also the type of vascular-attractive signal (or mixture of signals) being released. This primarily heterochronic and possibly chemically heterogeneous regional pattern would be diversely translated into different local amounts of VEGF-A or other signals at specific places and time-points, with eventual supra-threshold spiking that might elicit selective punctiform vascular responses (PVs) simultaneously at different places. Early molecular regionalization of the neural wall, plus additional modulating factors operating discretely at given ranges in the paraneural mesoderm (blocking early effect of SHH or other chordal signals), thus probably jointly impede a general wave of vascular penetration. Contrarily, molecular differential compartmentalization may restrict the highest capacity to trigger vascularization to discrete, spatially separated domains, which (each for different reasons) turn out to be relatively more favorable for vascular interactions, whereas other domains require more time to reach an equivalent status.

Another relevant factor causing heterogeneous vascular patterns may be represented at given sites by temporally heterogeneous formation of multiple disjoint PVVP fields, rather than a single all-encompassing PVVP wave, which was not observed in our material. The “outskirts” of these fields eventually may “violate” under appropriate circumstances some areal/zonal molecular boundaries, thus invading adjacent theoretically independent vascular domains. This might generate occasionally locally fused flux gradients between two or more neighboring histogenetic areas, allowing eventual formation of perforant periventricular arterial routes, as discussed above for the perforant thalamic vessels coursing periventricularly (*via* PVVPs; Figure 8C). In contrast, the short and long circumferential neuromeric arteries branching of from the basilar artery and reaching directly ventral and dorsal neuromeric parts of the hindbrain alar plate probably result from alternative condensation of blood flux *via* the perineural network (*via* PNVPs). Our data suggest anti-intuitively that the short mediobasal branches start to develop *later* than the correlative ventral and dorsal alar branches.

### Topology of Adult Human Arteries Relative to the Prosomeric Map

In the three aspects discussed above (PNVP, PVs, PVVP), we corroborated the initial hypothesis that embryonic brain vascularization progresses spatially and temporally in heterochronic and non-gradiental coordination with the spatially patterned molecular regionalization (and consequent differential histogenesis) of the brain wall. Notably, this occurs consistently with the prosomeric model, and not with the columnar model. Discrimination was found both along the DV axis (e.g., roof/alar/basal/floor differences) and the AP axis (e.g., neuromeric differences within the alar plate; absence of intraneural longitudinal vessels). Earlier columnar descriptions and interpretations of this complex pattern turned out to be less discriminative and did not reach similar conclusions. This difference was expected, since the columnar model does not accept neuromeres, and misinterprets diencephalic and hypothalamic neuromeres as longitudinal columns, due to its arbitrary forebrain axis definition (see Puelles, 2018, 2019, this book). The only subdivision principle available to Herrick, Kuhlenbeck and their followers was the column (theoretically thought to be *functionally* homogeneous—i.e., a column was held to subserve the same function along the whole brain, and therefore was not expected to show differential structural aspects along its length). The simplistic description by many columnar authors of a general wave of vascular invasion, thought to spread uniformly caudo-rostrally from a starting point at the lower medulla, probably was due to the poor capacity of their morphological paradigm to subdivide the hindbrain columns into smaller components (e.g., neuromeric units). As mentioned in the “Introduction” section, this unreal vascular wave propagation concept was paralleled by an equally simplistic propagation wave of neurogenesis postulated by other columnar authors up to the late 70s.

Subsequent neuromeric analysis, first without gene markers (e.g., Bergquist and Källén, 1954; Vaage, 1969; Puelles et al., 1987; review in Puelles, 2018), and later with them (e.g., Puelles and Rubenstein, 1993, 2003, 2015), demonstrated that overall proliferation or differentiation waves do not exist in the brain, due to the numerous interposed boundaries and the independent heterochronic behavior of small areal domains of the neural wall. We now know this is due to early transversal and longitudinal molecular patterning, which differentiates the neural wall into a checkboard pattern of well-delimited and molecularly diverse areas. Such compartments were first crudely recognized as “migration areas” by major neuromeric authors such as Bergquist and Källén (1954) or as “radial histogenetic units” (Puelles et al., 1987). These “fundamental morphogenetic units,” as they are called modernly (Nieuwenhuys and Puelles, 2016), autonomously regulate in a heterochronic manner their ulterior histogenesis according to their own unique gene activation profiles. A number of ulterior developmental processes possibly including vascularization (but also tangential cell migration and axonal navigation) proceed by appropriate reactions of moving cells or cell processes to molecular signals written out differentially at individual histogenetic units, either in the epitopic decoration of the cell membrane of radial glia and ventricular cells, or with a variety of molecules attached to neuronal membranes and to the intercellular matrix, or, alternatively, as molecules diffused gradientally within the local intercellular fluid. Such direct or indirect cell-cell interactions frequently generate polarization of the growing elements in longitudinal or transverse directions, when the interacting elements follow chemical traces shared particularly by longitudinal rather than neuromeric (transverse) developmental units, or vice versa. Since the histogenetic processes that construct the brain largely occur in its lateral walls (the floor and roof plates being rather quiescent regions), the distinction within these walls of transverse neuromeres and longitudinal basal and alar zones is particularly relevant (Figure 1A). Of course, finer subdivisions are also distinguishable as development advances (e.g., microzones or progenitor domains; see Puelles, 2013), but we have not found necessary to examine them at our present preliminary level of topologic analysis of the vascular pattern.

In the second part of this report, we accordingly addressed adult vascular patterns, knowing well that adult blood vessels not always reflect in their topography the early embryonic relationships they originally had relative to the invaded organ. However, the detailed mapping studies reported by Padget (1948, 1957) and Stewart (1955) for arteries and veins in the human brain offer considerable help, even though these authors hardly commented in this context on developmental units in the brain primordium. Since we know the deformed prosomeric regionalization pattern of adult rodent brains, we found it was possible to attempt tentative ascription of well described adult blood vessels to specific alar or basal penetration points and inner distribution fields within given unitary developmental blocks (neuromeres) of the forebrain and hindbrain Bauplan, or to particular courses of the subarachnoid arteries relative to the chessboard-like pattern of primary transverse and longitudinal boundaries deduced to exist intrinsically in the neural wall. The literature on brain vascular supply readily suggests that the pattern of human subarachnoid arteries is reproducible and not chaotic (and the same applies to other vertebrates studied). Leaving apart statistically minor variations, a number of constant features can be detected, which can be mapped with a degree of certainty with regard to the relative invariant position (topology) within our prosomeric model.

Our expectation was to find evidence that vessels named conventionally “anterior, middle, or posterior this or that” in adult neuroanatomy actually relate significantly in their subarachnoid and intraneural course with the underlying longitudinal and transversal partitions of the brain wall (Figure 1A). There is, of course, a before and an after to consider as the artery first approaches along a particular route and then penetrates the wall at a given neuromeric basal or alar area (e.g., collateral branches need to be considered as separate problems). In our present first approximation, we did not represent in high detail the vascular distribution within particular superficial or deep terminal fields, but terminal branches were expected *a priori* to remain largely within a given neuromeric and alar or basal areal unit of the brain wall. Two sorts of exceptions were observed in this regard, perforating thalamic vessels, and some rhombomeric arteries jumping into the cerebellum; the thalamic recurrent branch of the ach also may be inconsistent with the general model.

Given the tridimensional complexity of the object of study, we elaborated various sorts of schemata ranging from more realistic to more abstract ones, expecting them to jointly clarify our interpretation. The semi-realistic ones visualize the *human* brain arteries as represented upon a prosomeric brain map adapted from the simpler but homologous mouse brain morphology (we selected the human arteries, which in some aspects differ from the mouse ones, because of their higher practical interest for clinical readers; the mouse or rat pattern also would have served our general purpose but would have been less interesting). A prosomeric schema adapted to the shape of the human brain also is possible, but posed difficulties at the present stage due to the extensive deformations of the human brain and our poorer experimental knowledge of the corresponding developmental fate maps. In the next two sections, we will comment on our mappings of basal and alar plate arteries, which in many cases can be readily distinguished, with some exceptions. Along these sections, it will become apparent that restriction of most arteries to specific neuromeric territories is readily observable, with minimal exceptions to this rule.

#### Basal Plate Arteries

Throughout the brain, there is a numerous set of paramedian segmentally-restricted arteries that selectively supply the basal plate longitudinal zone, as defined by the prosomeric model (largely based on the old concept of His). We represented such basal plate arteries in our Figure 8A, consistently with the excellent sagittal injected photographic examples provided by Scremin and Holschneider (2012) and Scremin (2015) for rodents. Multiple paramedian basal branches are given out sequentially from the as, bas, pcer and pc vessels. These penetrate radially the basal zone in the topological transversal plane of the individual neuromeres (several branches per neuromere), as expected according to the observable incurvations of the brain axis, particularly at the cephalic flexure (see also mb; Figures 1C,D, 11). According to the literature, at medullary levels these radially penetrating basal vessels can be subdivided into “paramedian” and “lateromedial” arteries arising at the as and ve arteries (Salamon, 1971, 1973; Lazorthes et al., 1976; Ten Donkelaar, 2011). The alternative terms “mediobasal” and “laterobasal” arteries would be more explicit about the appropriate embryological and topologic ascription (mb, lb; Figure 1C). This duplication probably owes to the existence in the medullary area of two options, so that the as and ve basal branches “share” the basal plate distribution field. Note that alar populations tangentially migrated into the basal plate apparently get supplied by the local vessels. Similarly, basal neurons migrated tangentially into the alar plate are likewise served by the local alar branches. This suggests that their respective spatial selectivity refers to the neuromeric unit and to its basal/alar subdivision, but does not involve specific chemical contact-mediated recognition of basal or alar neurons.

Analogous medial vessels serving the segmental units of the forebrain basal plate sort out of the rostral end of the basilar artery or the initial course of the posterior cerebral artery, as *posteromedial central arteries*, as well as from the posterior communicating artery, *posterolateral central arteries* (Ten Donkelaar, 2011; his Figure 2.10). These arteries penetrate through the posterior perforated space, which, according to the prosomeric model, is diencephalic and in part hypothalamic—retromamillary—rostrally to the oculomotor root. The standard (columnar) neuroanatomy textbook version wrongly ascribes this space entirely to the midbrain. These forebrain mediobasal arteries irrigate topologically equivalent basal or medial portions of the midbrain, diencephalon and hypothalamic neuromeric territories (m1, m2; p1-p3; hp1-hp2). The longitudinal continuity of such basal plate vessels through the midbrain and diencephalic tegmentum into at least the retromamillary basal hypothalamus (Figure 11D), and not beyond, represents a pattern that corroborates the original basal plate concept of His (1895, 1904), which is maintained in the prosomeric model. In contrast, this pattern contradicts the columnar conception of the basal plate, which threats the whole hypothalamus including the preoptic area as the basal plate of the overlying diencephalon, and conceives likewise a telencephalic basal plate (Swanson, 2012). There clearly are no further basal plate medial arteries beyond the mamillary area (see also Scremin, 2015; his Figure 7).

This basal plate region is always strongly bent ventrally at the cephalic flexure, which causes these topologically radial arterial branches entering successive neuromeres to fan out in the sagittal plane (Figure 8A; neuromeric color code as in Figure 1A). As commented above, exceptionally some of these medial basal arteries—like the tuberculo-thalamic perforant artery from the pc, and inferior thalamic perforant artery from the pcer—spread their terminal branches beyond the basal plate proper into the suprajacent alar neuromeric domains (p2; alar thalamus domain). It is unclear in the literature whether this perforant pattern, which we related above to a possible pathway created *via* the PVVP networks, is a local peculiarity of p2, or something non-exceptional for the forebrain. We need specific analysis of this point.

#### Alar Plate Arteries

##### Hindbrain

Individual or double alar plate arterial branches for each of the hindbrain neuromeres are given out by the vertebral and basilar arteries. These alar arteries are usually designated “short or long circumferential arteries,” particularly when originating from the basilar artery. This name refers to their external dorsal course circumventing the basilar pontine gray or the olivary bulges of the basal medulla, before entering their alar targets. An alternative terminology sensitive to the alar topography of their penetration sites and terminal fields might be “ventral alar” and “dorsal alar” arteries. Some of the alar segmental branches are short and penetrate the ventral or liminar sector of the alar plate (close to the alar-basal border), whereas others are longer and invade more extensive dorsal parts of the hindbrain alar plate, eventually supplying as well chorioidal roof plate branches (mainly described for the cerebellar arteries). It should be noted that fate-mapping studies have shown that each rhombomere possesses its own band of chorioidal roof (e.g., Marín and Puelles, 1995); it might be expected, thus, that all segmental dorsal alar arteries give out a chorioidal branch at their end.

The sc, aic and pic cerebellar arteries are special cases of such alar branches, since, *after* having performed their alar segmental role, they invade the cerebellum, escaping the neuromeric rule [they originate at isthmic (or r0), r5 and r9 levels and jump from there into r1, the main cerebellar site]; it is unclear why a major cerebellar artery does not arise directly at r1 level. Apparently, no pic artery reaches the cerebellum in the mouse, where the corresponding r9 branch of the vertebral artery remains a standard dorsal alar branch (Scremin and Holschneider, 2012).

A collateral point that merits passing comment is that the ventral *alar* hindbrain domain that is served by the short circumferential “*ventral alar*” neuromeric arteries singularly includes in its mantle layer, apart the somatosensory/viscerosensory columns and associated lateral reticular formation, the *branchiomotor and visceromotor cranial nerve nuclei*, as can be readily observed in the detailed schemata shown by Ten Donkelaar (2011) as his Figures 2.26c (motor trigeminal nucleus) and 2.27a–c (facial, ambiguus, dorsal vagus nuclei). This fact probably confused previous authors about the real position of the alar-basal boundary, and deterred them from realizing that these short circumferential arteries are selective *alar plate arteries*, similarly as anteromedian (mediobasal) and anterolateral (laterobasal) basilar segmental branches are typically *basal plate arteries*. In general, it can be noticed that neuroanatomic terminology for arteries does not use the concepts *alar* and *basal*, nor the descriptors *transversal* vs. *longitudinal*, which figure so prominently as objective patterns consistent with molecular genoarchitecture in our topologic prosomeric analysis of early steps in PNVP, PV and PVVP formation. The explanation for the cited mixing of basal visceromotor and branchiomotor brainstem nuclei with alar locations is known since the work of Ju et al. (2004). These originally basal-derived motor populations translocate their somata tangentially into the ventral part of the alar plate at intermediate developmental stages. This migratory process had already been visualized by various authors in the seventies and eighties (e.g., Windle, 1970), but had not been recognized as finishing inside the ventral or liminar alar plate. Ju et al. (2004) benefitted from a molecular delimitation of the alar-basal border to reach the correct conclusion. This result was recently corroborated in the mouse, using transgenic specific labeling of all alar-derived hindbrain neurons (Puelles et al., 2018; see this reference for a full review of this topic). There are comparative grounds to believe this is a general process in vertebrates, with few species variants (Nieuwenhuys and Puelles, 2016). Accordingly, the migratory interpretation can be extrapolated as well to the human brainstem. This represents a clearcut case where alar arterial branches serve basal neuronal populations that migrate into their alar plate territory.

##### Midbrain

There are also distinct arteries that penetrate directly the midbrain alar plate, though these data invariably refer to m1 (the rostral midbrain prosomere or mesomere), and we have no data at all about the recently recognized mesomere 2, whose alar domain is now known as “preisthmus” (an inconspicuous area lying caudal to the inferior colliculus, but rostral to the isthmic hindbrain; see reviews in Puelles et al., 2012; and Puelles, 2013, 2016). Some authors merely mention “mesencephalic” arteries, without further precisions about alar vs. basal distribution (e.g., Padget, 1948). The “quadrigeminal artery” described by Haines (1991, 1997) in the human brain clearly is an alar midbrain vessel (quad; Figure 10). It is probably identical with the “transverse collicular” artery cited as an early branch of the rodent pcer by Scremin (2015). This author mentions that its distribution ends at the brachium of the inferior colliculus and neighboring part of the inferior colliculus, which possibly indicates a *ventral alar* nature. On the other hand, multiple *dorsal alar* arteries penetrate radially both colliculi, originating in rodents from a “supracollicular plexus,” probably derived also from the pcer, which apparently anastomoses with overlying cortical vascularization (Scremin, 2015). This would represent another violation of the rule restricting neuromeric branches to specific neuromeres. Some sources also mention collicular branches stemming from the superior cerebellar artery, which is topologically isthmic in its initial course (sc; Figure 8B); this also would violate the said rule.

##### Diencephalon

Irrespective of confusing aspects due to poor resolution in the literature of the diencephalic vascular complexity, comparison of the pcer system of vessels with the prosomeric model has led us to realize that, interpreted topologically, the pcer course after it emerges from the basilar artery cannot be really “posterior,” as it seems at first glance. After passing rostral to the oculomotor nerve, the artery first moves transversally out of the floor plate-related median position of the basilar artery, to a position lateral to the pes pedunculi, roughly at the level of the pretectum (the mes-pretectal border lies just in front of the oculomotor nerve root; pcer; Figure 8B). This is possibly equivalent to a dorsoventral locus just above the alar-basal boundary, i.e., in a ventral part of the alar plate. This locus manifestly lies at the caudal part of the diencephalon, and from there the pcer necessarily must approach *longitudinally and rostralwards* the telencephalon (pcer; Figures 1A, 8B, 9A–C). To be able to approach the telencephalon, it must course successively along the lateral wall of the neuromeric pretectal, thalamic and prethalamic alar diencephalic regions, which undoubtedly lie rostral to the midbrain, and caudal to the telencephalic pallium (Figures 1A, 8B). In its P2 segment, the pcer is thus essentially a longitudinally coursing alar forebrain vessel giving rise successively to alar branches to midbrain, pretectum, thalamus and prethalamus (Figures 1A, 8B, 9A). The existence in humans of multiple transverse alar diencephalic branches of the pcer is well documented (e.g., Salamon, 1971, 1973). The pcer also contributes doubly to the diencephalic chorioidal roof plate domain in its thalamic and prethalamic sectors (posteromedial and posterolateral choroid branches, respectively), but not to the telencephalic chorioidal sector, served by the anterior chorioidal artery (Figures 8B,C, 9A–D, 10).

We have thus realized that diencephalic alar vascularization relates importantly to the longitudinal diencephalic courses of: (1) the pcer, rostralward along its P2 segment; (2) the *recurrent thalamic artery* or rth (we propose this new term), caudalward from the ach (the rth is conventionally named also “ach,” though this particular recurrent branch does not relate at all with chorioidal plexi); and (3) partly, to the pc and its perforant thalamic branches (Figures 8B, 9, 10). All three of these sources are in principle able to produce sequentially branches that enter separately the three diencephalic prosomeres (p1-p3; pretectum, thalamus, prethalamus). The pcer P2 segment and the rth branch of the ach course along the diencephalic alar plate, while the pc lies under the basal plate (pcer; rth; pc; Figure 10); the latter only reaches the thalamus and potentially other alar diencephalic domains—prethalamus, pretectum?—*via* perforant branches ingressed *via* the basal plate. Surprisingly, the longitudinal topology of these three sources of diencephalic branches had not been emphasized before in the relevant literature. Our Figure 9 schemata aim to explain in particular why the longitudinal pcer segment does not seem to be longitudinal in the adult.

Padget (1948, 1957) and Stewart (1955) mentioned “diencephalic” arteries and veins, but did not attend to possible neuromeres, and did not recognize either that these vessels were selective alar or perforant alar branches. This probably occurred because the columnar paradigm prevalent at the time understood the thalamus as a floating egg intercalated between striatum and midbrain, lacking any basal/tegmental correlate. The true diencephalic tegmentum (see Figures 1A, 8A) was instead given over to the midbrain/hypothalamus pair, thought to be mutually continuous, thus causing long-standing erroneous beliefs about the midbrain nature of the whole interpeduncular fossa and of the whole substantia nigra and ventral tegmental area, both of which are plurisegmental and mesodiencephalic (Puelles et al., 2012b; Puelles, 2013, 2016, 2019 this book). Actually, the columnar thalamus initially contained dorsal and ventral moieties (Herrick, 1910; Kuhlenbeck, 1973), but many authors using the columnar paradigm thought it to be simpler to refer to the whole egg-shaped mass as an unit. This distorting view still emerges in modern sources such as the Allen Mouse Brain Atlas (adult version). The simplified egg-shaped mass compounding thalamic and prethalamic elements is the terrain whose vascularization has been actively investigated, to the exclusion of any other diencephalic portion (e.g., the work of Tatu et al., 1998, 2001 and others reproduced in Ten Donkelaar, 2011 includes the prethalamic reticular nucleus as a thalamic component). Thus, diencephalic vascularization became largely simplified to just “thalamic” vessels, with some weakly connected hypothalamic asides (because the columnar model expects the hypothalamus to be a basal and floor part of the diencephalon, a point that we already have shown the basal plate vessels do not corroborate). In conclusion, the subject of alar diencephalic vascularization merits a thorough reexamination consistent with the modern prosomeric approach, to clarify relevant details pertaining to its individualized pretectal, thalamic and prethalamic neuromeric territories, and clearly distinguish from them the separate and relatively more rostral alar hypothalamic territory, which needs an analysis connected instead with *telencephalic* vascularization (only very modestly attempted in our Figure 10, due to lack of precise data).

The alar vascularization of the pretectum is wholly undescribed so far. This is no doubt due to the Cinderella-like character it gained as a consequence of the columnar tradition to *not recognize* this territory as a straightforward neuromere distinct from the thalamus in front and the midbrain at the back (see contrary prosomeric evidence and discussion in Ferran et al., 2008; Puelles, 2013, 2016, 2018; Nieuwenhuys and Puelles, 2016). Columnar authors figuratively swept the pretectum under the carpet by arbitrarily dividing pretectal derivatives into caudal ones ascribed to the midbrain and rostral ones ascribed either to the epithalamus or to the dorsal thalamus. We have shown in the first part of our “Results” section that the mouse alar pretectum has a characteristically precocious early vascularization pattern that is independent from thalamus and midbrain. It seems thus reasonable to predict that some of the alar “thalamic” branches of the pcer actually are alar “pretectal” branches (symbolically represented by branches with a question sign in p1; Figures 8B, 9, 10). It is hoped that future research will cover this weak spot in vascular neuroanatomy. It would be of particular interest to know whether some of the pretectal basal plate arteries extend as “pretectal perforant” arteries into the overlying alar domain. Moreover, it may be expected that specific arterial branches so far undescribed serve the secretory subcommissural organ at the pretectal roof plate.

Recorded description of thalamic vascularization is in contrast quite advanced, having been systematized into five arterial pedicles worked out by several authors (Lazorthes et al., 1962, 1976; Plets et al., 1970; Percheron, 1976a,b, 1977; Tatu et al., 1998, 2001): (1) tubero-thalamic perforant artery from the pc; (2) inferior thalamic perforant from the pcer; (3) inferolateral thalamo-geniculate vessels from the pcer; (4) pulvinar branches from the plch artery of the pcer; and (5) geniculate branches from the ach (internal carotid). Of these, numbers 1 and 2 are perforant vessels that reach the alar thalamus *via* the underlying basal plate, presumably entering selectively through the p2 tegmentum (e.g., Salamon, 1971, his Figure 33; Haines, 1997; his Figure 14.14). We already advanced above the hypothesis that such extraordinary perforant courses (Figure 8C) may perhaps be explained by the observed peculiar retardation of PVs appearance in the thalamic alar field (practically none found still at E11.5), and the possible emergence of alternative PVVP pathways spreading into the thalamus from more precociously-formed basal plate PVVP in the same neuromere. We also reached the conclusion that the habitual descriptions of the tubero-thalamic artery as being related at its entry point to optic chiasma and tuberal or tubero-mamillary (or premamillary/mamillary) areas, and supposedly serving the mamillothalamic tract and the prethalamic reticular nucleus, seem inconsistent with all the drawings published of this artery, which consistently show it arising from the pc midway along its “diencephalic tegmental” course between the ic and the pcer. There is no inner logic, nor any actual evidence, as far as we can tell, for derivation of branches from this perforant artery under the thalamus into distant rostral hypothalamic areas (three neuromeres away; Figure 10). We think we may have here a semantic error due to faulty ascription of the “tubero-” root instead of the proper “tuberculo-” root to the name of this artery. As accepted by tradition, the “tubero-thalamic perforant” concept implies the assumption of a false premamillary general position and a false tuberal terminal field. It is on the other hand possible that analysis of the historic sources of this term may yet reveal that the original expression (perhaps in Latin?) was “tuberculo-thalamic perforant artery,” referring to a different tuberosity. Indeed, in classic comparative neuroanatomic works what we now identify as thalamic and prethalamic tegmentum was referred to as “posterior tuberculum,” meaning that it produced a bulge into the ventricular surface at the posterior end of the third ventricle. Our analysis therefore proposes a credible “*tuberculo-thalamic perforant artery*” in place of the doubtful “tubero-thalamic” notion. Other branches of the pc possibly may supply the tuberal and tubero-mamillary basal plate regions, and a separate (and so far undescribed) perforant pc branch given at p3 level may serve the reticular nucleus and other alar prethalamic derivatives.

Numbers 3 and 4 among the thalamic pedicles cited above are direct alar arteries derived from the pcer P2 segment along its longitudinal course lounging the primitive alar diencephalic lateral surface. The thalamo-geniculate arteries may divide into external pretectal, thalamic and prethalamic branches, unless there exist completely separate pcer arteries for each of these alar diencephalic territories (Figure 9). Given that the posteromedial and posterolateral chorioidal arteries of the pcer finish at the roof plate and nearby hyperdorsal alar thalamic sites, such as the habenula and the associative thalamic nuclei (laterodorsal, lateral posterior, pulvinar), they may perhaps be considered to represent dorsal alar thalamic arteries, whereas the thalamo-geniculate arteries may represent ventral alar thalamic vessels, particularly if they also serve the topologically more ventral thalamic nuclei. The ventralmost thalamic nucleus is the medial geniculate body (Puelles, 2001). However, this division into ventral and dorsal alar vessels may be an unnecessary extrapolation into the diencephalon of brainstem patterns.

The issue of prethalamic vascularization is hardly covered at all by the literature so far (but Stewart, 1955 maps the “thalamic” chorioidal vessels relative to the well characterized ventral thalamus, zona limitans and dorsal thalamus, wrongly understanding them as columnar elements rather than as neuromeric ones). A distinct prethalamic part of the diencephalic wall placed *rostral* to the presumptive mammalian thalamus was already depicted by Ziehen (1906) and Bailey (1916), before Rendahl (1924) first defined the zona limitans boundary (see reviews in Puelles, 2018; and also Puelles, 2019; this book). The unfortunate prevalence of Herrick (1910) columnar model already in this period somehow dispersed any interest in that objective topologic relationship, which made diencephalic “columns” impossible (compare Swanson, 2012 “columnar” persistence in the negation of the zona limitans, against substantial recent molecular and experimental evidence). Present analysis is expected to stimulate researchers disposing of relevant material to re-examine it with the compressed and tridimensionally deformed prethalamic region in mind (Figure 9). Our present analysis of the marked morphogenetic deformation of the prethalamus (covered also in Puelles, 2019, this book) follows, corroborates and expands earlier classic examples, notably those of Schwalbe (1880) and Hochstetter (1895, 1898, 1919).

The present dearth of data on the prethalamic vascularization pattern may result clarified by pointing out that the posterolateral chorioidal artery (pcer system) seems essentially a prethalamic dorsal alar artery (consistently with data of Padget, 1948, 1957 and Stewart, 1955). Indeed, it targets the dorsalmost alar prethalamus (the prethalamic eminence) and the associated chorioidal plexus (the post-foraminal part of the lateral ventricle chorioidal formation; see Puelles, 2019, this book). Other prethalamic arteries may come from the inferolateral or thalamo-geniculate arteries (pcer system), as was suggested above, and/or from the anterior chorioidal artery (either *via* its *recurrent thalamic* branch, as it approaches the lateral geniculate body, or *via* its *subpallial collateral branches* to the internal pallidum and innominate area, which lie quite close to the reticular nucleus; Puelles, 2019, this book). Note in this latter regard that if we interpret at face value the dotted *central* territory of the ach in Figures 2.8, 2.9 of Ten Donkelaar (2011; extracted from Tatu et al., 1998, 2001; see also Lazorthes et al., 1962, 1976), this territory abuts directly the ovoid thalamus mass, precisely along its limit with the prethalamic reticular nucleus, which is thus implied to fall within the ach territory (perhaps unwittingly). At more dorsal section levels (such as Ten Donkelaar’s correlative Figure 2.7) it is the mcer central distribution field in the *striatum* which abuts directly the thalamic ovoid (note it was wrongly believed in older times that the thalamus contacts the striate body), implying that the corresponding adjacent upper part of the prethalamic reticular nucleus might be served by the mcer *via* its lateral striate or pallidal branches. However, if we examine the relative positions of the mcer territory and the prethalamus in our Figure 10, it seems doubtful that a striatal mcer branch can reach the reticular nucleus, or the immediate vicinity of the thalamus. Such branches would have to cross the interposed pallidal and diagonal subpallial domains. It is more probable that Lazorthes et al. (1962, 1976), Percheron (1977), Tatu et al. (1998, 2001) and Ten Donkelaar (2011) may have unwittingly forgotten the intercalated prethalamic (i.e., ventral thalamic) position of the reticular nucleus (plus some other grisea), and been led astray by the old assumption that the striatum contacts directly the thalamus at the opto-striate sulcus, a postulate that is untenable nowadays (see Puelles, 2019, this book). In that case, we need a better informed corroboration of the exact relationship of the branches of the ach or mcer arteries with the prethalamic reticular nucleus and other prethalamic derivatives.

In addition, the literature stating that our *tuberculo-thalamic perforant artery* (the conventional tubero-thalamic artery; tth; Figure 8D) serves also the reticular nucleus (Tatu et al., 1998, 2001) suggests either collateral thalamo-prethalamic branches crossing the interthalamic zona limitans, or a parallel prethalamic perforant artery either from the posterior communicating artery, or from the root of the tth.

The plch artery and its corresponding fissural plexus portion (Figures 8D, 9, 10; see below) clearly seems related to the prethalamic taenia, which normally is not described in the adult brain (though embryos clearly show it; see Bailey, 1916; Puelles, 2019, this book). On the other hand, the anterior chorioidal artery, a direct and early developing branch of the internal carotid artery (Padget, 1948), serves the sphenoidal part of the same chorioidal plexus, irrigating also the tail of the caudate nucleus and the tail of the bed nucleus striae terminalis, among other targets (see below). Padget (1957) identifies separate veins for these two chorioidal masses of the lateral ventricle. This gives added weight to the argument affirming that the supracapsular fissural chorioidal sector whose apparent taenia lies along the thalamic chorioidal sulcus is actually prethalamic, while only the sphenoidal sector is fully telencephalic (according to the prosomeric model, there *must* be a prethalamic chorioidal sector intercalated between the thalamic and telencephalic chorioidal formations; Figure 10). Figures 9, 10 further explain and complement our conclusions on these chorioidal arteries, together with the widely admitted notion that the posteromedial chorioidal artery, also a branch of the pcer, serves selectively the thalamic median chorioidal plexus of the third ventricle.

##### Secondary Prosencephalon

The present topologic analysis of the vascular pattern at the secondary prosencephalon is also mapped in Figure 10. The terminal branches of the ic—acer, mcer—are not problematic. We see the anterior communicating artery as a longitudinal alar vessel that contributes to serving the preoptic and overlying septal regions. The oph and sh arteries are also longitudinal vessels (alar and basal, respectively). The pc artery apparently contributes significantly to the basal hypothalamic field (Ten Donkelaar, 2011; Scremin, 2015), though this is somewhat at odds with Salamon (1973) thesis that the rostral part of the diencephalic tegmentum (substantia nigra and peduncle), lying caudal to the basal hypothalamus, is served by the ach (from the internal carotid). In general, one may conclude that the vascularization of the hypothalamus requires additional research within our present model.

## Ethics Statement

All mouse experiments were approved by the ethical committee from the University of Murcia.

## Author Contributions

The work was planned by JF and LP. RM-M and PM-O carried out the experiments, with technical help from AA. All authors analyzed and discussed the data. LP, AV, and JF wrote the manuscript.

## Conflict of Interest Statement

The authors declare that the research was conducted in the absence of any commercial or financial relationships that could be construed as a potential conflict of interest.

## Footnotes

^1^developingmouse.brain-map.org

## Glossary

A: amygdala
A1: anterior cerebral artery segment 1
A2: anterior cerebral artery segment 2
ABB: alar-basal boundary
ac: anterior communicating artery
acer: anterior cerebral artery
ach: anterior chorioidal artery
aco: anterior commissure
AH: adenohypophysis
aic: anteroinferior cerebellar artery
AP: anteroposterior axis or dimension
ap: alar plate
ATHy: acroterminal hypothalamus
Atd: acroterminal domain
as: anterior spinal artery
bas: basilar artery
calc: calcarine artery
Cb: cerebellum
cc: corpus callosum
chp: chorioidal plexus
chf: chorioidal fissure
cmarg: callosomarginal artery
CoP: commissural pretectum
bp: basal plate
da: dorsoalar artery
dbc: decussation of brachium conjunctivum
Dg: diagonal domain
DMB: diencephalo-mesencephalic boundary
DTu: dorsal tuberal region
DV: dorsoventral axis or dimension
EP: eye primordium
F: frontal lobe
fcht: fissural chorioidal tela
fp: floor plate
fpol: frontopolar artery
Hb: Hindbrain
hc: hippocampal commissure
Hy: hypothalamus
hp1: hypothalamo-telencephalic prosomere 1
hp2: hypothalamo-telencephalic prosomere 2
ic: internal carotid artery
if: interventricular foramen
Isth: Isthmus
IC: inferior colliculus
Ins: insular lobe
JcP: juxtacommissural pretectum
lb: laterobasal artery
LG: lateral geniculate body
LGE: lateral ganglionic eminence
LP: lens placode
m1: mesomere 1
m2: mesomere 2
mam: mamillary body
mb: mediobasal artery
mb: mediobasal branches P1 segment posterior cerebral artery
Mb: midbrain
mcer: medial cerebral artery
MGE: medial ganglionic eminence
NH: neurohypophysis
OC: optic cup
on: optic nerve
OP: olfactory placode
oph: ophthalmic artery
orb: orbitofrontal artery
OS: optic stalk
ot: optic tract
OV: optic vesicle
P: parietal lobe
p: pons
p1: prosomere 1
p2: prosomere 2
p3: prosomere 3
P1: posterior cerebral artery segment 1
P2: posterior cerebral artery segment 2
P3: posterior cerebral artery segment 3
P4: posterior cerebral artery segment 4
Pall: pallidum
pao: parieto-occipital artery
pc: posterior communicating artery
pcer: posterior cerebral artery
PcP: precommissural pretectum
pec: pericallosal artery
ped: peduncle
PHy: peduncular hypothalamus
Pi: pineal gland
pic: posterior inferior cerebellar artery
plch: posterolateral chorioidal artery
pmch: posteromedial chorioidal artery
POA: preoptic area
PT: pretectum
PTh: prethalamus
pthchf: prethalamic chorioidal fissure
PNVP: perineural vascular plexus
PV: perforant vessels
PVVP: periventricular vascular plexus
quad: quadrigeminal artery
r0: rhombomere 0
r1: rhombomere 1
r2: rhombomere 2
r3: rhombomere 3
r4: rhombomere 4
r5: rhombomere 5
r6: rhombomere 6
r7: rhombomere 7
r8: rhombomere 8
r9: rhombomere 9
r10: rhombomere 10
r11: rhombomere 11
Rh: rhombencephalon
rh-chp: rhombencephalic chorioidal plexus
rm: retromamillary region
rp: roof plate
rth: recurrent thalamic artery
SC: superior colliculus
sc: superior cerebellar artery
sco: supracollicular arterial network
SCor: spinal cord
Se: septum
sh: superior hypophyseal artery
SP: subpallium
St: striatum
tchf: telencephalic chorioidal fissure
Tel: telencephalon
temp: temporal artery
tgd: midbrain dorsal tegmental decussation
Th: thalamus
th-chp: thalamic chorioidal plexus
thg: thalamo-geniculate artery
Tu: tuberal region
va: ventroalar artery
ve: vertebral artery
ZLI: zona limitans intrathalamica
THy: terminal hypothalamus
thp: thalamo-perforant artery
tth: tuberculo-thalamic artery (commonly misnamed “tubero-thalamic”)
